# Therapeutic treatment of hepatitis E virus infection in pigs with a neutralizing monoclonal antibody

**DOI:** 10.1038/s41598-025-95992-x

**Published:** 2025-03-28

**Authors:** Isabella Hrabal, Elmira Aliabadi, Sven Reiche, Saskia Weber, Cora M. Holicki, Laura Schmid, Christine Fast, Charlotte Schröder, Benjamin Gutjahr, Patrick Behrendt, Martin H. Groschup, Martin Eiden

**Affiliations:** 1 Institute for Novel and Emerging Infectious Diseases, Friedrich-Loeffler-Institut, Greifswald – Insel Riems, Germany; 2 Institute for Experimental Virology, Centre for Experimental and Clinical Infection Research, TWINCORE, Hannover, Germany; 3 Helmholz Center for Infection Research GmbH, Braunschweig, Germany; 4 Department of Experimental Animal Facilities and Biorisk Management, Friedrich-Loeffler-Institut, Greifswald – Insel Riems, Germany; 5 Institute of Diagnostic Virology, Friedrich-Loeffler-Institut, Greifswald – Insel Riems, Germany; 6 Department of Viroscience, Erasmus Medical Center, Rotterdam, The Netherlands; 7 Institute of Molecular Virology and Cell Biology, Friedrich-Loeffler-Institut, Greifswald – Insel Riems, Germany; 8 Department of Gastroenterology, Hepatology, Infectious Diseases and Endocrinology, Hannover Medical School, Hannover, Germany; 9 German Centre for Infection Research, Partner site Braunschweig-Hannover, Braunschweig, Germany; 10 German Centre for Infection Research, Partner Site Hamburg-Lübeck-Borstel-Riems, Greifswald – Insel Riems, Germany

**Keywords:** Hepatitis E virus, Therapy, Monoclonal antibody, *In vitro* characterization, Pig model, Virology, Medical research, Experimental models of disease, Translational research, Infectious diseases, Diseases, Hepatitis, Liver diseases

## Abstract

Hepatitis E virus (HEV) poses a significant risk to human health. In Europe, the majority of HEV infection are caused by the zoonotic genotype 3 (HEV-3), which can cause chronic hepatitis E in immunocompromised patients and those with pre-existing liver disease, and may eventually develop into fatal liver cirrhosis. In this study, we examined the effectiveness of a monoclonal antibody (MAb) treatment strategy using a well established HEV-3 pig model with intravenous infection. For this purpose, nine MAbs raised against the viral capsid protein were generated and the neutralizing activities were compared using in vitro assays. The antibody with the highest neutralizing activity, MAb 5F6A1, was selected for an in vivo study in pigs infected with HEV-3. Following the initial infection of pigs with HEV-3, MAb 5F6A1 was administered intravenously one and seven days post-infection. The results suggest MAb 5F6A1 significantly reduced viremia and virus shedding in pigs infected with HEV-3. This study provides significant insight into the dynamics of HEV infection in pigs and highlights the efficacy of MAb based therapy as an option for treating HEV in porcine hosts and, potentially, humans.

**Supplementary Information:**

The online version contains supplementary material available at 10.1038/s41598-025-95992-x.

## Introduction

Hepatitis E virus (HEV) is an agent of significant public health concern, considered to be the leading cause of acute viral hepatitis^1,2^. HEV is primarily transmitted through the fecal-oral route, and typically associated with contaminated water and food^3^. The HEV species *Paslahepevirus balayani* (family *Hepeviridae*) includes several genotypes that are known to be pathogenic to humans^4^: genotypes 1 and 2 (HEV-1 and HEV-2) are found solely in humans; and genotypes 3 and 4 (HEV-3 and HEV-4) circulate between animal reservoirs and humans^5^. While HEV-3 is widespread in Europe, reports of HEV-4 are relatively rare. However, the prevalence of HEV-3 in Europe has been steadily increasing, attracting more attention in recent years^6^. The virus in maintained in nature by pigs and wild boars^7–9^, though wild rabbits have also been identified as potential reservoirs^10^. Effective control and management of HEV transmission requires a comprehensive One Health approach, but this has been hindered by lack of awareness, underdiagnosis and challenges in surveillance^11–13^.

The virus can manifest as both acute and chronic infections. While acute HEV-3 infections typically follow a silent or asymptomatic and self-limiting course, there is an increased risk of developing a chronic HEV infection with persistent HEV viremia in people with pre-existing liver damage or in patients who are undergoing immunosuppressive therapy due to underlying diseases. Of the chronically infected patients, approximately 15% fail to clear the infection^14^. This failure can be attributed to factors such as insufficient efficacy of medication, side effects leading to discontinuation of medication, virus mutations or contraindications due to underlying diseases^15–17^. Thus, additional treatment options are urgently needed for chronically infected patients to prevent HEV-related morbidity and mortality.

Over the last three decades, monoclonal antibodies (MAbs) have emerged as a potential treatment option for a wide range of infectious diseases^18^. MAbs, due to their ability to target specific epitopes, low potential for side-effects, and their capacity to enhance immune responses, represent a potential alternative for managing chronic HEV infections in patients for whom current pharmaceutical treatments, as recommended by the European Association for the Study of Liver Disease (EASL) guidelines, are ineffective or contraindicated^16,19,20^. To date, there have been no therapeutic applications of MAbs for the treatment of HEV. Here, we measure the effect of MAbs on viral replication and shedding in pigs infected with HEV-3.

## Results

### Development, characterization and epitope mapping of monoclonal antibodies against HEV-3 capsid protein

**Fig. 1:**
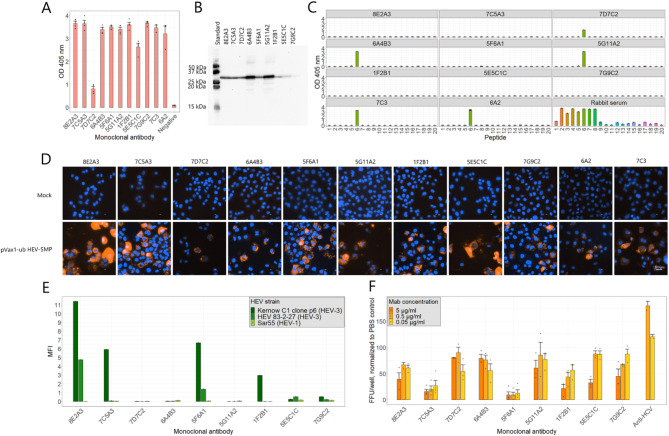
Results of in vitro characterizations of MAbs. (**A**) ELISA of Mabs. Hybridoma cell culture supernatants containing MAbs were tested against partial capsid protein p239 (HEV-3) coating antigen for their presence of capsid targeting MAbs. Two in-house MAbs, 7C3 and 6A2, that are directed against the HEV capsid protein, were used as positive controls (data unpublished). (**B**) Western blot analysis for determining the specificity of MAb supernatants against p239 protein. To evaluate the specificity of multiple MAbs simultaneously, the membrane was split and processed separately for each Mab, then reassembled for imagine (**C**) Peptide ELISA of MAbs in cell culture supernatants. Overlapping peptides, covering the complete HEV p239-region, were used as coating antigens. Two in-house MAbs, 7C3 and 6A2, which are directed against the HEV capsid protein, as well as serum of a rabbit that was vaccinated with partial capsid protein p429 (Supplemental Data 1) with detection reagent, were used as positive controls. (**D**) Immunofluorescence assay of supernatants in non-transfected mock control (top) and pVax1-ub HEV-SMP transfected cells (bottom). Two in-house MAbs, 7C3 and 6A2, that are directed against the HEV capsid protein, were used as positive controls. (**E**) Immunofluorescence staining of cells transfected with Kernow C1 P6 and pUC83-2 in a concentration of 5 µg/ml. The analysis was performed using an ELISpot Reader (Immunospot, Shaker Hights, Cleveland, UA). A monoclonal anti-HCV antibody was used as a negative control. (**F**) Neutralization activity of monoclonal antibodies against non-enveloped HEV-3 strain Kernow C1 p6 in serial dilutions, starting at a concentration of 5 µg/ml. An anti-HCV antibody and phosphate buffered saline (PBS) were used as negative controls. Data were normalized to corresponding PBS control. Depicted are the means and standard error of measurements.

After vaccinating mice with purified, recombinant capsid protein p239 (HEV-3, Material and Methods 4.2^21^). , and generating hybridoma cell lines, we identified nine hybridoma cell clones that produced MAbs with detectable reactivity against the p239 in an enzyme-linked immunosorbent assay (ELISA) (Fig. 1A). The binding specificity of these MAbs to the capsid protein was confirmed by Western blot, where all nine MAbs demonstrated selective reactivity against p239 (25.6 kDa) (Fig. 1B, Supplemental Fig. 2).

To determine whether the antibodies reacted to specific HEV peptides within the capsid protein, a peptide ELISA was conducted using overlapping peptides of the viral open reading frame (ORF) 2 segment coding for the p239 partial capsid protein (Fig. 1C, Supplemental Table 1). In this assay, three monoclonal antibodies—5G11A2, 6A4B3 and 7D7C2—exhibited binding to a specific linear peptide sequence (peptide 6, QDKGIAIPHDIDLGDSRVVI, amino acid position 421–440), while the remaining six antibodies did not interact with peptides.

The binding specificity of MAbs to a more native conformation of the viral capsid was assessed via indirect immunofluorescence assays. Therefore, human hepatoma PCL/PRF/5 cells were transfected with a plasmid (pVax1-ub HEV-SMP) expressing the S, M and P domains of HEV ORF 2 (gene sequence presented in Supplemental Data 1). Using this approach, all nine monoclonal antibodies targeting the capsid protein successfully detected ORF 2 protein domains in transfected cells (Fig. 1D), with 7D7C2 showing a weaker signal in this assay compared to the other monoclonal antibodies.

Additionally, binding to two different HEV-3 strains, “Kernow C1 clone p6” and “HEV83-2-27” (pUC83-2-27), as well as to HEV-1 strain Sar55, was evaluated in transfected HepG2/C3A human hepatocellular carcinoma cells (Fig. 1E). In this assay, antibodies were used from hybridoma culture supernatant. Six of the nine antibodies (8E2A3, 7C5A3, 5F6A1, 1F2B1, 5E5C1C and 7G9C2) were able to recognize Kernow C1 clone P6, with 8E2A3, 7C5A3 and 5F6A1 exhibiting the highest mean fluorescence intensity (MFI) values (above 5). In contrast, strain PUC83-2-27 was recognized by four monoclonal antibodies (8E2A3, 5F6A1 and 5E5C1C and 7G9C2), with 8E2A3 showing the highest MFI, followed by 5F6A1 (Fig. 1E). The HEV-1 strain, Sar55, was not detected by any MAb. Images of immunofluorescence are displayed in the Supplemental Fig. 3.

The neutralization capacity of MAbs against the non-enveloped form of HEV-3 Kernow C1 clone p6 was examined using a 10-fold dilution series starting at concentrations of 5 µg/ml (Fig. 1F). All MAbs exhibited a neutralization activity in a concentration of 5 µg/ml. In lower concentrations, 7C5A3, 5F6A1 and 1F2B1 demonstrated the highest neutralization capacity, indicated by the lowest number of focus forming units (FFU) per well. Additional dilutions of those antibodies are accessible in the supplementary material (Supplemental Fig. 4), confirming that 5F6A1 was the best performing MAb in the neutralization assay. Finally, the antibody isotype was determined as Immunoglobulin G (IgG) subtype 1 with a kappa (κ) light chain.

### Treatment of infected pigs with monoclonal antibody 5F6A1 and evaluation of antibody titers in ELISA

**Fig. 2:**
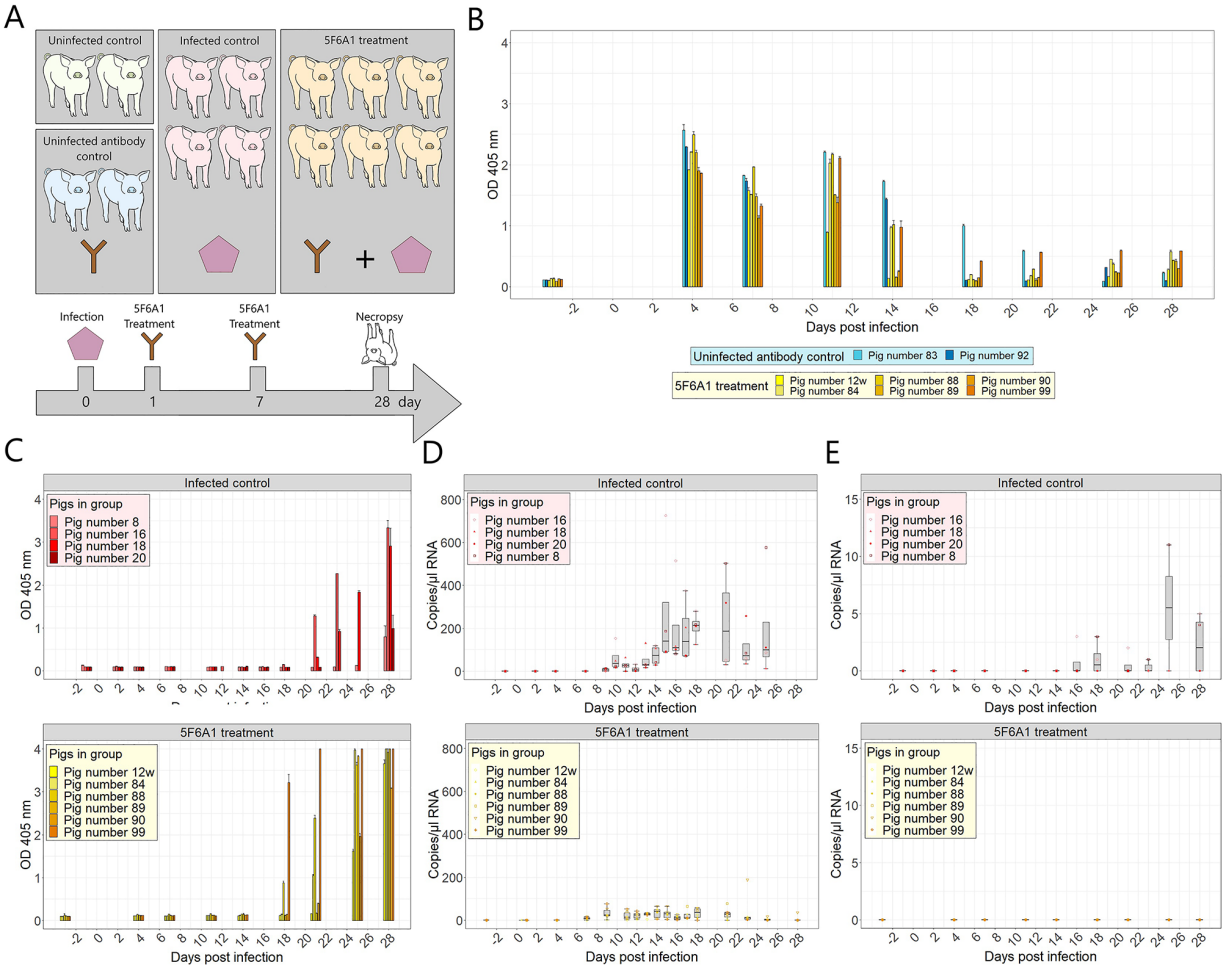
The HEV treatment experiment in pigs. (**A**) Overview of the experimental design in the 5F6A1 treatment experiment. (**B**) Partial HEV capsid protein p239 ELISA using rabbit anti mouse antibody as secondary antibody for detection of applicated murine mAb 5F6A1. Samples were run in duplicates. Depicted are the means and standard error of measurements. (**C**) Partial HEV capsid protein p239 ELISA using protein G as the secondary antibody for detection of swine antibodies after infection. Samples were run in duplicates. (**D**) HEV-3 RNA in fecal samples, detected by RT-qPCR. (**E**) HEV-3 RNA in serum samples, detected by RT-qPCR. Note the different scaling of the y-axes in comparison to “D”. Bars depict mean values. Error bars depict the standard error. Box-and-whisker plots show mean and interquartile range.

Based on the in vitro results, MAb 5F6A1 was selected for therapeutic application in a pig infection model. For this purpose, the antibody was purified as shown in Supplemental Fig. 1A. Six pigs were intravenously infected with 2 ml of a HEV-3 inoculum (9.5 × 10^3^ copies/µl). Each pig then received a dose of purified MAb 5F6A1 (2.46 g/dose) one day and seven days post-infection. Serum samples were collected twice a week and assessed for antibody development/generation against HEV using ELISA (Fig. 2A). Blood chemistry parameters were analyzed in serum samples collected before infection and at necropsy (Supplemental Table 2). The administered antibodies were specifically determined with a modified p239 ELISA, using rabbit anti-mouse antibodies as a secondary antibody (Fig. 2B), which exclusively binds to murine antibodies. Using this approach, circulating MAbs were detectable in pigs from the 5F6A1 treated group as well as in the uninfected antibody control group until day 11, which is four days after the second MAb administration. The applied antibody remained detectable for up to seven days in three out of four pigs following the second administration. This included one pig from the uninfected antibody control group and two pigs from the 5F6A1 treatment group. Data from the untreated animals are shown in the Supplemental Fig. 5C.

Assessment of HEV-3-specific antibody development in response to the infection involved both an ELISA targeting p239 (Fig. 2C, Supplemental Fig. 5B), and an ID Screen^®^ Hepatitis E Indirect Multi-species ELISA (ID.vet Innovative Diagnostics, Grabels, France, Supplemental Fig. 5A). The ID.vet ELISA showed an increase in serum antibodies in all infected animals of the control group from 21 days after infection with the HEV inoculum. An increase of antibodies in the treated group was only observed from day 25 post-infection. In contrast, the ELISA with p239 coating antigen revealed an increase of antibodies targeting p239 already at day 18 post-infection in the treated animals, while antibodies in untreated animals were detectable only from day 21 (Fig. 2C).

### Evaluation of viral load in feces, serum and organ samples

Virus shedding via feces and virus circulation in serum were monitored by RT-qPCR throughout the infection study. In all four animals of the untreated infection group, virus shedding via feces was detected from day 9 and 10. In animals treated with the monoclonal antibody 5F6A1 post-infection, virus shedding began at day 4 (pig number 99) or day 7 (all other pigs of the treatment group) (Fig. 2D). However, the treated animals shed a significantly lower amount of the virus compared to the untreated animals. Viral RNA was detectable in serum samples from two of four untreated control pigs (pig number 8 and pig number 16). In contrast, none of the pigs in the treated group exhibited any detectable viral RNA in their sera (Fig. 2E).

After necropsy, samples of the cranial mesenteric lymph node, spleen, liver, bile, gallbladder wall, brain and kidney were examined for the presence of viral RNA. From the control group, viral RNA was detected in the bile of all animals. Additionally, for two of the four pigs, RNA was also detected in the spleen and liver. HEV RNA could also be detected in the gallbladder wall of one pig. Conversely, in the mAb-treated group, viral RNA was only detected in the bile, and at lower RNA copy numbers, except for one outlier (pig number 90) (Table 1).

**Table 1:** Viral load in organ samples after necropsy.

Group	Animal ear tag number	Viral load in tissue [copies/µl RNA]						
		Brain	Liver	Gallbladder wall	Spleen	Kidney	Cranial meseneteric lymph node	Bile
Uninfected control	Number 17	–	–	–	–	–	–	–
	Number 19	–	–	–	–	–	–	–
Uninfected antibody control	Number 83	–	–	–	–	–	–	–
	Number 92	–	–	–	–	–	–	–
Infected control	Number 8	–	456.4	238.9	1.9	–	–	890,100
	Number 16	–	5.3	–	3.2	–	–	24,290
	Number 18	–	–	–	–	–	–	81.88
	Number 20	–	–	–	–	-	–	406.4
Antibody treatment group	Number 12 white	–	–	–	–	–	–	8.8
	Number 84	–	–	–	–	–	–	1.5
	Number 88	–	–	–	-	–	–	–
	Number 89	–	–	–	–	–	–	15.7
	Number 90	–	–	–	–	–	–	12,000
	Number 99	–	–	–	–	–	–	–

### Statistical analysis

The obtained data are positive skewed distributed. For statistical evaluation of our data, two test procedures (Wilcoxon Rank Sum Test) was performed. A p-value less than 0.05 was considered significant. A significant difference in fecal viral load excretion was observed between the infected control group and the MAb-treated group (*p* < 0.01). A significant difference was also observed in the serum viral loads between the control and treated group (t-test: *p* < 0.05, Wilcoxon Rank Sum Test: *p* < 0.01).

Furthermore, the Wilcoxon Rank Sum Test indicated significant differences in viral load in the liver, gallbladder wall, spleen (*p* < 0.1), and bile (*p* < 0.05) between the control and treatment groups. An overview of exact p-values for each test is listed in the Supplemental Table 3.

## Discussion

MAbs have proven effective in treating and preventing a diversity of viral infections^22^, offering a promising, highly specific and potent therapeutic approach with minimal side effects^19^. Several MAb applications have been established and demonstrated their ability to improve clinical outcomes for both acute and chronic viral infections^23–26^.

Several MAbs targeting the HEV capsid protein have been developed so far. Some have shown promising results in preventing HEV infection in cell culture, reducing viral shedding or providing partial protection against infection in rhesus monkeys and rabbits^27,28^. Despite these advancements, no MAb has received clinical approval for use in humans. The pig infection model, used here, shares significant physiological and immunological similarities with humans, making them an ideal model for studying the virus-induced immune response and pathogenesis. Additionally, pigs are large enough animals to allow for comprehensive virological and immunological analyses. The combination of physiological, immunological and genetic parallels to humans make pigs an important tool for translational biomedical research^29^. Compared to other animal models, they have substantial advantages over mouse models. For example, mice are not naturally susceptible to HEV-3 infection, therefore, humanised or immunocompromised mice are needed, though they may not closely mimic natural infection in humans. Although rabbits are naturally susceptible to rabbit HEV, this HEV species genetically distinct from the zoonotic porcine HEV-3 genotypes, potentially limiting translational relevance. Non-human primates offer the greatest physiological and immunological similarity to humans, but are ethically challenging^30^.

In this study, we analyzed nine novel mouse-derived MAbs targeting the HEV-3 partial capsid protein p239. The p239 protein mirrors the sequence of Hecolin^®^ (Xiamen Innovax Biotech Co., Xiamen, China), the only available vaccine against HEV so far, that is based on a HEV-1 strain. Hecolin^®^ has been established as an effective vaccine against HEV-4 in China for over a decade^31^ and its ability to induce neutralizing antibodies in humans has been demonstrated before^32^. A previous study suggests, that the corresponding HEV-3 derived p239 vaccine, which was used for generation of MAbs in this study, is able to induce partial protection by preventing virus shedding in HEV-3 infected pigs^33^, making it a suitable protein for generation of HEV-targeting MAbs.

The nine MAbs used here were assessed for their binding capacity to capsid fragments through ELISA, Western blot and immunofluorescence assays, as well as their capacity to neutralize HEV-3. Among them, three MAbs (5G11A2, 6A4B3 and 7D7C2) exhibited specific binding to the linear peptide sequence QDKGIAIPHDIDLGDSRVVI (amino acid position 421–440 of the capsid protein). Tang et al. (2015)^34^ A previous study stated, that antibodies targeting the motif KGIAIPHDIDLG (aa 423–437), within the same linear peptide sequence, were able to block virus entry of non-enveloped HEV-1 and HEV-4 into HepG2/C3A cells in vitro. However, it has been observed that this epitope is not immunodominant during natural HEV infection progression, and antibodies generated against this epitope post-vaccination seem to have only partial protective effects in vivo^34^. Despite their capacity to identify the linear peptide, none of these three antibodies displayed enhanced efficacy in neutralizing activity in this study either, indicating that the peptide might not be accessible within the complete virus particle.

MAb 5F6A1 exhibited specific binding activity to partial capsid proteins in PLC/PRF/5 cells and demonstrated strong reactivity with the two HEV-3 strains, pUC83-2 and Kernow C1 clone P6 in HepG2/C3A cells. In addition, it exhibited the highest neutralizing capacity with lowest IC_50_ values for non-enveloped viral particles of Kernow C1 clone P6 in HepG2/C3A cells. Based on these findings, MAb 5F6A1 was selected for assessment in a pig infection model as a potential therapeutic option against HEV-3. For this purpose, we administered a dose of 2.46 g MAb to pigs one- and seven-days post-infection with a liver-derived inoculum. Some pigs sporadically developed a slight fever up to a maximum of 39.7 °C during the experiment (clinical score sheets, Supplemental Data 2 and Supplemental Table 4). During the subsequent blood sampling, monoclonal mouse antibodies were detected in pig serum up to seven days post administration.

Only two of the four untreated infection controls displayed detectable viremia. The inability to detect HEV in the blood of all pigs is not unexpected and can be attributed to the transient nature of viremia in HEV infections, which typically lasts only 1–2 weeks^35^. This brief duration makes the timing of detection critical. Furthermore, viremia is considerably shorter than liver infection or fecal shedding, both of which last longer and often contain significantly higher levels of viral RNA^33,36,37^. Despite the limitations of using viremia as a parameter to evaluate the efficacy of antibody therapy, our study revealed significant findings: In contrast to the untreated animals, treated animals showed no detectable viremia, indicating that the administered MAb may have effectively neutralized circulating viral particles or reduced their release into the bloodstream through currently unknown mechanisms.

Despite this absence of detectable viral RNA in the serum, viral RNA remained detectable in feces and bile of treated pigs. This may be due to the limited accessibility of viral particles to the administered MAb. Following infection, viral particles are likely localized within the cytoplasm of infected cells^38^, but the access of monoclonal antibodies to these intracellular regions is likely to be limited after intravenous administration^39^. Consequently, MAb monotherapy may not fully neutralize viral particles once cells are already infected with HEV. Nevertheless, Fc-receptor functions, such as antibody-dependent cellular cytotoxicity (ADCC), antibody-dependent cellular phagocytosis (ADCP) or complement-dependent cytotoxicity (CDC) could still play a role, potentially contributing to the significantly reduced viral load observed in the feces and bile compared to untreated pig^40,41^. Of particular note was the observation that the entire infection and recovery process, including the time of antibody formation against p239 and the beginning and the end of virus shedding, appeared to be shifted forward in the treated animals compared to the infected control group. This could also be the reason for the absence of virus detection in any organs other than the bile in treated animals, as virus shedding had already ceased at the time of necropsy, in contrast to untreated animals.

One factor that contributes to this earlier appearance of signs of infection could be the induction of an complement or Fc-receptor mediated antibody-dependent enhancement effect by the administered antibodies^42^. A previous study indicated, that HEV-3 strains are able to infect human derived monocytes and macrophages in vitro^43^. The infection of these immune cells is known to have an major impact on the occurrence of those effects^44^. The presence of antibodies directed against HEV, especially subtherapeutic concentrations, might therefore have resulted in an earlier uptake of viral particles into monocytes and macrophages, and consequently a shifted onset of immune responses and viral shedding^45^, especially when taking previous reports of cross-species reactivities of Fc-receptors (FcRs) and antibodies into account^46,47^.

The binding of the Fc domain of antibodies to corresponding FcRs on immune cells triggers specific effector responses. These include antibody-dependent cellular cytotoxicity (ADCC) through FcR binding on natural killer cells, antibody-dependent phagocytosis (ADCP), mediated by Fc receptors on monocytes and macrophages and complement-dependent cytotoxicity (CDC) driven by interactions with complement factors. Together, these mechanisms contribute to the clearance of cells expressing foreign antigens^48^. The murine IgG1 5F6A1 shows promising potential to induce ADCP, as cross-species binding between murine IgG1 and human FcγRIIA and FcγRIIB has been reported^49^. Similarly, human IgG1 has been shown to bind to murine FcγRIII and FcγRIIB, as well as to orthologous porcine FcγRIIA and FcγRIIB^47,50,51^. The high conservation of the Fc-binding domain across orthologous Fc receptors in mice, humans, and pigs suggests that murine IgG1 may also exhibit affinity for porcine FcγRIIA and FcγRIIB. The targeting of these receptors on monocyte populations is further evidence of their possible involvement in ADCP, which corresponds to their functional role in humans^48,51^.

Additionally, weak cross-species complement activation by murine IgG1 in the presence of human complement has been observed, indicating a potential role for CDC in the pig treatment experiment^52^. Studies also suggest that murine IgG1 can bind to the porcine neonatal Fc receptor (FcRn), enabling IgG transcytosis from the bloodstream into the intestinal lumen. This mechanism may allow 5F6A1 to neutralize non-enveloped viral particles effectively^53,54^.

Other effects, such as immunogenicity-enhancement, should be considered as a reason for the earlier signs of viral shedding. Host sex may also play a role, as the treatment group consisted solely of male piglets, while the control group included both males and females due to random selection. However, statistical analysis showed no significant impact of sex on viral shedding within the control group (Supplemental Table 3). Therefore, further studies should be conducted to minimize the risk of adverse effects in case of a therapeutic use of the MAb.

Despite the observed effects in treated pigs, the mechanisms behind the reduction of viral particles remain unclear. HEV particles circulating in the blood are known to be enveloped by host-derived lipid membranes, forming quasi-enveloped HEV (eHEV). Because these particles are covered by this membrane, antibodies targeting the capsid are unlikely to bind effectively to the enveloped form of the virus^55^. In addition, the role of T-cell response in controlling HEV infection has to be further elucidated^56,57^. It may also be important to evaluate the treatment at a later time point, e.g. starting from day 7 post-infection rather than day 1. Delayed treatment could provide valuable insights into sustained therapeutic efficacy and future studies should explore this approach to better reflect the natural course of infection.

Despite these open questions, we demonstrate that the administration of the antibody 5F6A1 to infected pigs resulted in a significant reduction of viral load in blood, feces and tissues compared to untreated pigs. This finding makes 5F6A1 a promising therapeutic candidate for treating acute and chronic HEV-3 infections in humans, particularly when existing drug treatments are ineffective.

## Materials and methods

All experiments were performed under Biosafety level 2 (BSL2) conditions.

### Animals

Experiments with animals were performed in accordance with national and European regulations. Authors complied with the ARRIVE guidelines 2.0 for reporting of conditions and findings of animal-connected experiments.

Two *BALB/c* mice originated from the Friedrich-Loeffler-Institute’s internal mouse breeding program and were housed in local facilities. The experiment was conducted in line with the national and European legislation (EURL 63/2010), with approval by the s Federal State of Mecklenburg-Western Pomerania, Germany (file number: 7221.3-2-042/17).

Additionally, 14 commercial hybrid pigs were procured from a local breeder (Landboden Glasin, Glasin, Germany) at the age of 10–14 weeks and a weight of 25–35 kg. The swine experiment and sampling protocol was approved by the competent authority of the Federal State of Mecklenburg-Vorpommern, Germany, prior to its implementation based on national and European legislation, EURL 63/2010 for the protection of laboratory animals (reference 7221.3-1-010/22). No additional preregistration was arranged. A statistical evaluation was carried out to determine the group size in accordance with national legislation section 31, Paragraph 1 of the *Tierschutzversuchstierverordnung* (Animal Welfare Ordinance). The treatment group size was determined using G*Power 3.1.9.4^58^ to achieve a statistical power of 80% with a significance level of 5%.

Animals were acclimated for three days in the local husbandry of the Friedrich-Loeffler-Institut prior to infection. Throughout the experiment, all possible measures were implemented to enhance overall well-being of the pigs, to create an enriched environment for the pigs and to keep the number of animals in the experiment to the required minimum.

### Proteins used in the study: synthesis and purification

To generate MAbs, a recombinant bacterial protein, derived from a plasmid encoding the partial HEV-3 capsid protein p239, was used as an immunogen. The plasmid, synthetized as part of an earlier study^21^, encodes a 239 amino acid (aa) segment of the HEV-3 capsid protein, corresponding to nucleotide positions 6300–7016 of strain KP294371. The protein was also used as an antigen for antibody detection and to determine binding specificity.

The recombinant capsid protein was expressed in *E. coli* BL21(DE3) (Thermo Fisher Scientific, Life Technologies GmbH, Darmstadt, Germany) and subsequently purified via Ni-NTA columns, following previously established procedures^59^ (Qiagen manual, Qiagen, Hilden, Germany), under denaturation conditions, with an elution buffer pH adjusted to 5.9 and 4.5. Following purification, proteins underwent dialysis in a 0.05 M carbonate-bicarbonate buffer (pH 10.3).

Purity of the recombinant protein was determined using sodium dodecyl-sulfate polyacrylamide gel electrophoresis (SDS-PAGE), as previously described^21^ (Supplemental Fig. 1B).

Quantification of protein concentration was performed using Roti^®^Quant universal kit following the manufacturer’s instructions (Carl Roth GmbH + Co. KG, Ebersberg, Germany). The resulting protein was stored at − 20 °C.

#### Mouse immunization and cell fusion

Fifty µg of bacterially expressed protein p239 was mixed with an equal volume of GERBU Adjuvant MM (v/v) (Biotechnik Gerbu, Heidelberg, Germany) and administered intraperitoneally into *BALB/c* mice at 4-week intervals. After four vaccinations, the mice were euthanized by inhalation of an isoflurane overdose followed by intracardial exsanguination. Spleen cells from the mice were fused with SP2/0 myeloma cells, and the resulting hybridoma cells were screened for monoclonal antibodies against HEV-3 capsid protein using an indirect enzyme linked immunosorbent assay (ELISA). Stabile hybridoma clones were established as described elsewhere^60^.

### Purification of antibodies

The antibodies were purified using HiTrap Protein G antibody purification columns (GE Healthcare Bio-Sciences, Uppsala, Sweden) according to the manufacturer’s instructions. The elution fractions were dialyzed against phosphate buffered saline (PBS) pH 7.4, and the protein concentration was determined colorimetrically using Roti^®^Quant universal (Carl Roth GmbH + Co. KG, Ebersberg, Germany) as outlined in the user’s manual. Purity of the MAb before and after dialysis was determined by SDS-PAGE (Supplemental Fig. 1A). The MAb for the treatment experiment was classified according to the Mouse Monoclonal Antibody Isotyping Test Kit (Bio-Rad, Feldkirchen, Germany) following the manufacturer’s instructions. The MAbs were centrifugated and supernatant was aliquoted into 0.88 ml portions, with a concentration of 2.65 mg/ml, and stored at − 20 °C until use.

### ELISA

An indirect antigen ELISA was performed according to standard protocol^59^ using recombinant partial HEV capsid protein HEV p239^33^. Nunc MaxiSorp 96-well plates (Invitrogen by Thermo Fisher Scientific, Life Technologies GmbH, Darmstadt, Germany) were coated with the corresponding antigen in a concentration of 1 µg/ml in 0.05 M carbonate-hydrogen carbonate buffer pH 10.3. Following a blocking step with 10% skimmed milk in PBS, cell culture supernatants, containing MAbs, were added 1:2 in 2% skimmed milk powder, or serum samples were added 1:25, and incubated for one hour at 37 °C.Two MAbs (7C3 and 6A2) against the HEV capsid protein were used as positive controls (Laboratory Eiden, Friedrich-Loeffler-Institute, Greifswald – Insel Riems, Germany, data unpublished). After washing with PBS/0.1% Tween, horseradish peroxidase (HRP) conjugated polyclonal Rabbit Anti-Mouse Immunoglobulins (1:2000, Dako, Hamburg, Germany) or Protein G, HRP conjugate (1:5000, EMD Millipore Corp., Darmstadt, Germany) was applied as secondary antibody. A solution of 2,2’-azino-bis-3-ethylbenzothiazoline-6-sulphonic acid (ABTS) was then added and the plates were incubated for 30 min at room temperature. The reaction was then stopped with 1% sodium dodecyl sulfate (SDS), and the optical density (OD) at 405 nm was measured in an ELISA-reader (Tecan, Männedorf, Switzerland). The MAb used in the treatment experiment was classified according to the Mouse Monoclonal Antibody Isotyping Test Kit (Bio-Rad, Feldkirchen, Germany) following the manufacturer’s instructions.

A commercial ELISA (ID Screen^®^ Hepatitis E Indirect Multi-species, ID.vet Innovative Diagnostics, Grabels, France) was also performed following the ID Screen manual.

### Peptide ELISA

An ELISA assay using biotinylated oligopeptides, each 20 amino acids long with a 12-amino acid overlap (produced by JPT Peptide Technologies GmbH, Berlin, Germany), was used to identify linear epitopes of the newly generated antibodies. Corresponding sequences, covering 239 amino acids of the central ORF 2 capsid protein, are depicted in the supplementary material (Supplemental Table 1). The peptides were dissolved in 100 µl dimethylsulfoxide (DMSO) and stored at − 20 °C. The peptides were then diluted 1:250 in coating buffer (PBS-0.05% Tween, 40% DMSO) and 100 µl of the solution was added to 96-well streptavidin plates (Nunc Immobilizer Streptavidin, ThermoScientific, Life Technologies GmbH, Darmstadt, Germany) and incubated for one hour. After washing the plates four times with PBS-0.05% Tween, a two-step blocking procedure was performed: First, a 30-minute blocking with blocking buffer I (PBS, 20% sucrose, 0.4% biotin) at room temperature, followed by a one hour blocking with blocking buffer II (3% Bovine serum albumin (BSA) in Tris buffered saline (TBS) 0.1% Tween) at 30 °C. Subsequently, undiluted antibody-containing cell culture supernatants were incubated on the plate for one hour at 30 °C. After three washes with TBS-0.05% Tween, mouse anti-rabbit-HRP (diluted 1:2000 in blocking buffer II) was added and incubated for one hour at 30 °C. The plate was washed three times with TBS-0.05% Tween and ABTS was added. After 30 min the reaction was stopped with 50 µl 1% SDS and the OD was measured (as above). The two HEV capsid directed MAbs 6A2 and 7C3 (above) and serum from a rabbit vaccinated with a partial HEV capsid protein (p429, Supplemental Data 1) were used as positive control. The rabbit serum was diluted 1:25, and Protein G, functioning as a detection reagent, was used at a 1:5000 dilution.

### SDS-PAGE and Western blot

SDS-PAGE were made using 13% or 16% bis/acrylamide gel as described earlier^59^. Staining was conducted using ROTI^®^ Blue quick (Carl Roth GmbH + Co. KG, Ebersberg, Germany) according to manufacturer’s instructions. For Western blot analyses, protein samples were transferred from SDS-PAGE to Polyvinylidene fluoride (PVDF) membranes (Immobilon^®^, Carl Roth GmbH + Co. KG, Ebersberg, Germany) by semi-dry electroblotting. After blocking with blocking buffer (PBS, 0.1% Teen, 5% sim milk powder) at room temperature for 30 min, membranes were incubated for one hour in the corresponding primary antibody, diluted 1:20 in blocking buffer. As negative controls, three monoclonal antibodies, directed against HEV-ORF 3 (Laboratory Eiden, Friedrich-Loeffler-Institute, Greifswald – Insel Riems, Germany, data unpublished), or a Rift Valley fever - virus (RVFV) directed MAb^61^ were used. 6A2 and 7C3, two in-house HEV capsid protein directed antibodies (data unpublished) were utilized as positive controls (Supplemental Fig. 2). After washing with PBS-0.1% Tween, the membrane was incubated in HRP-conjugated polyclonal Rabbit Anti-Mouse Immunoglobulins (1:2000), and visualized using chemiluminescent substrate SuperSignal™ West Pico Plus (ThermoScientific, Life Technologies GmbH, Darmstadt, Germany) according to the manufacturer’s instructions on a Versa.Doc Imaging System (Bio-Rad Laboratories, Hercules, California, USA).

### Indirect Immunofluorescence assay (IFA)

Sterile 96-well tissue culture plates (Falcon, Fisher Scientific, Life Technologies GmbH, Darmstadt, Germany) were seeded with 3 × 10^5^ PLC/PRF/5 cells in Eagle’s minimum essential medium (EMEM) supplemented with 10% heat-inactivated fetal calf serum (FCS), 2 mM l-glutamine, 1% non-essential amino acids (NEAA), 100 U/mL penicillin G, 100 µg/mL streptomycin, 2.5 µg/mL amphotericin B (Sigma laboratories, Mumbai, India) and 30 mM MgCl_2_ (MEMM) at 37 °C and 5% CO_2_^62^. After 24 h incubation the confluency reached about 80–90%. Medium was replaced by Opti-MEM (Zellbank, Friedrich-Loeffler-Institut, Greifswald – Insel Riems, Germany) medium containing 100 U/mL penicillin G, 100 µg/mL streptomycin and 2.5 µg/ml amphotericin B and cells were transfected with the pVax1-ub HEV-SMP plasmid, containing the HEV-3 ORF 2 S, M and P domains (GenBank accession number KP294371, Supplemental Data 1). Transfection was performed using Lipofectamine^®^2000 (Invitrogen by Thermo Fisher Scientific, Life Technologies GmbH, Darmstadt, Germany) according to manufacturer’s instructions. After a growth period of 3 days at 37 °C and 5% CO_2_, cells were fixed with 100 µl 4% paraformaldehyde (PFA, Carl Roth, Ebersberg, Germany) for 30 min at room temperature and permeabilized with 50 µl Triton X 0.1% for ten minutes. Blocking was performed in two consecutive steps: Incubation with 0.1 M 50 µL Glycin solution 0.1 M (Carl Roth GmbH + Co. KG, Ebersberg, Germany) for ten minutes, and with 50 µl blocking reagent (2% BSA, 0.2% Tween, 3% glycerin and 0.5% sodium acid) for 30 min.

For staining, wells were incubated with cell culture supernatants containing monoclonal antibodies (0.5 mg/ml, diluted 1:10 in blocking reagent) for one hour, washed and finally incubated with Cy3™-conjugated AffiniPure Goat Anti-Mouse IgG (H + L) (Jackson ImmunoResearch, West Grove, Pensilvania, USA) and DAPI (1:20000, Invitrogen by Thermo Fisher Scientific, Life Technologies GmbH, Darmstadt, Germany) for one hour. The two MAbs, directed against the HEV capsid protein (7C3 and 6A2), were used as positive controls, as described before. Wells were washed three times with 50 µl PBS and staining was evaluated by fluorescence microscopy (Nikon, Tokyo, Japan) using the 40 × 0.6 objective and exposure times of 40 ms (DAPI) or 140 ms (Cy3).

To evaluate the binding of MAbs to different HEV-3 strains, human hepatoma HepG2/C3A cells were transfected with HEV constructs according to the protocol described before^63^. Two different plasmids were employed for the transfection, one encoding the full-length HEV-3 “Kernow-C1 clone p6” (GenBank accession Nr.: JQ679013), and the other encoding the “HEV83-2-27 clone” (GenBank accession Nr.: AB740232). Additionally, a plasmid containing the subgenomic sequence of Kernow-C1 p6 HEV linked to a Gaussia luciferase reporter gene, and the HEV-1 strain (“Sar55”, GenBank accession Nr.: AF444002.1) were used as controls. The transfection was carried out via electroporation. Five days after transfection, the cells were fixed with 3% PFA in PBS and permeabilized with 0.2% Triton. Cells were then incubated overnight at room temperature with MAb. In this assay, antibodies were used from hybridoma culture supernatant. A monoclonal anti-hepatitis C virus (HCV) antibody served as negative control. The following day the cells were stained using Alexa Fluor 488-conjugated anti-mouse IgG. Images were captured at 10x magnification using an Olympus IX2 inverted microscope (Olympus, Tokyo, Japan), and analyzed using ImageJ software^64^ to calculate the MFI.

### Neutralisation assay

MAbs were titrated in duplicate in ten-fold serial dilutions, starting from a concentration of 5 µg/ml, in EMEM low IgG FCS medium. Naked HEV genotype 3 viral strain Kernow C1p6 G1634R was prepared according to the previously established protocol^63^. Each MAb dilution (40 µl) was combined with 40 µl of medium containing the naked HEV-3 and incubated at 37 °C for one hour. Parallel assays were performed with PBS and an anti-HCV antibody as negative controls. The MAb-virus mixtures were then applied to human hepatoma cells (HepG2/C3A) that had been seeded in 96-well plates, and incubation was continued at 37 °C with 5% CO_2_. After 24 h, 100 µl of fresh medium was added. Four days post-infection, the cells were fixed with 3% PFA in PBS and permeabilized with 0.2% Triton. Cells were subsequently stained for the ORF2-encoded capsid protein. Focus-forming units (FFUs) were counted using an ELISpot reader (Immunospot, Shaker Hights, Cleveland, UA). The endpoint FFU in the presence of MAb was calculated as: 100 × [average FFU in the presence of MAb / average FFU in the presence of PBS].

### Inoculum and infection procedure

A 25% (w/v) liver inoculum was prepared from a liver sample sourced from a wild boar experimentally infected with HEV-3^37^ (GeneBank Accession Nr. KP294371.1). The inoculum contained not only naked but mostly enveloped virus particles, which was experimentally confirmed by sucrose density gradient fractionation^65^. Liver tissue was homogenized in PBS using a TissueLyser II (Qiagen, Hilden, Germany), followed by centrifugation at 7,459x*g* for five minutes. The supernatant was pooled and filtered twice through syringe filters (0.22 µl Millex^®^-GP 33 mm filter unit, Carrigtwohill, Ireland). The infectious homogenates were aliquoted into 2 ml portions and stored at − 80 °C until use. The corresponding inocula were thawed over night at 4 °C and acclimated to room temperature shortly before infection. In pigs, the infection was administered intravenously into the cranial vena cava. Intravenous inoculation provides a standard infection model, that is used also in other pig infection studies with similar infection courses and patterns^37,66,67^.

### Molecular analysis

From the fecal samples, a 25% (w/v) suspension was prepared using a 0.89% NaCl solution. The suspension was thoroughly mixed then centrifuged at 7,500 x g at 4 °C for ten minutes. 100 µg of tissue samples were homogenized in 500 µl of ZB5 cell culture medium, containing MEM and NEAA (Zellbank, Friedrich-Loeffler-Institut, Greifswald – Insel Riems, Germany) using a TissueLyser II (Qiagen, Hilden, Germany), followed by centrifugation. Serum samples were obtained by centrifugation of blood samples at 833 x g for 12 min. The pellets were discarded.

Genomic material was extracted from 100 µl of serum, inoculum, or supernatant from homogenized tissue or feces, using the NucleoMag VET kit (Macherey-Nagel, Düren, Germany) according to manufacturer’s instructions, in combination with the KingFisher Flex extraction robot (ThermoScientific, Life Technologies GmbH, Darmstadt, Germany).

Viral RNA quantification was performed using quantitative real-time reverse transcription-polymerase chain reaction (qRT-PCR) for the ORF 2/3 overlapping region of HEV-3 following an established protocol^68^ on a BioRad CFX96™ Real-Time System. β-actin and MS2^69^ were used as RNA extraction controls. For quantification, standards were generated through digital droplet PCR (ddPCR) using the One-Step RT-ddPCR Advanced Kit for Probes 200rxns (Bio-Rad Laboratories, Hercules, California, USA) and the Bio-Rad QX-200^®^ Droplet Reader (Bio-Rad Laboratories, Hercules, California, USA). The HEV-3 RNA used for the standards was obtained from the liver tissue of an experimentally infected wild boar^37^. The samples were classified as HEV-negative if the PCR results showed a copy number per microliter (copies/µl) below 1 compared to the standards.

### Experimental design of the pig treatment study

Fourteen piglets (10–14 weeks, 25–30 kg) were randomly selected by animal keepers from a commercial fattening farm in Germany (Landboden Glasin, Glasin, Germany). Before infection, fecal samples and serum from all piglets were tested for previous exposure to the HEV-3 pathogen using RT-qPCR and ELISA. During the experiment, daily monitoring of clinical parameters, general condition and behavior was conducted, with a clinical score recorded. The evaluation form, the list of observed parameters, and the criteria for termination of the examination are listed in Supplemental Data 2.

The experiment design, including infection procedure and sampling, was based on previously established protocols^33,37^. The animals were divided into four groups: uninfected control (*n* = 2), infected control without antibody treatment (*n* = 4), uninfected control with antibody control (*n* = 2), and an infected treatment group (*n* = 6) (Table 2). The animals were housed in separate stable units containing two to four pigs of the same group. Organic chemistry of blood samples, taken before infection and 28 days post-infection, was conducted using the Vetscan V2 device and the comprehensive diagnostic profile (Abraxis, Zoetis, Parsippany-Troy Hills Township, New Jersey, USA, Supplemental Table 2).

The pigs in the treatment group and the infection control group were intravenously infected with 2 ml inoculum (9.6 × 10^3^ copies/µl) on day zero. Subsequently, piglets in the treatment group, as well as the animals in the uninfected antibody control group, received doses of purified monoclonal antibodies (2.46 g/dose) on day one and day seven.

Blood and fecal samples were collected before infection and every 2–3 days post-infection. From day ten to day 20 post-infection, fecal samples were obtained on a daily basis.

Four-weeks post-infection, the animals were euthanized by electrical stunning followed by exsanguination. Necropsies were conducted and samples from blood, feces, bile and tissues were aliquoted for RNA extraction and stored at − 80 °C. Infection, treatment and sample analyses were performed by the same examiners. Therefore, no additional blinding measures could be taken.

**Table 2:** Overview of pigs in the experiment.

Group	Number of animals	Gender	Infection	Treatment	Animal identification (Eartag number)
Uninfected control	2	Male Female	No	No	Number 17 Number 19
Uninfected antibody control	2	Male Male	No	Yes	Number 83 Number 92
Treatment group	6	Male Male Male Male Male Male	Yes	Yes	Number 12 white Number 84 Number 88 Number 89 Number 90 Number 99
Infected control	4	Female Male Female Female	Yes	No	Number 8 Number 16 Number 18 Number 20

In general, yellow ear tags were used to identify the pigs, except for pig 12, which had a white ear tag and was therefore referred to as “12 white (12w)”.

### Statistical analysis

The statistical analysis of the animal test data was performed using the R statistical software (R Core Team [2023]. _R: A Language and Environment for Statistical Computing_. R Foundation for Statistical Computing, Vienna, Austria). Data were evaluated for normality using Shapiro-Wilk test, qq-plot and histogram. Differences between treatment groups were examined using a Welch two-sample t-test and the Wilcoxon Rank Sum Test. In the context of the t-test, the null hypothesis (H0) proposed that the mean of the antibody treatment group exceeded that of the infected control group, while the alternative hypothesis (H1) suggested the contrary: H0 = µ_treatment_ ≥ µ_infected control_; H1 = µ_treatment_ < µ_infected control_. The code and data used for statistical analysis are available in the Supplemental Data 3 and the Supplemental Table 5.

## Electronic supplementary material

Below is the link to the electronic supplementary material.

Supplementary Material 1

## Data Availability

All data generated or analyzed during this study are included in this published article and its Supplementary material files.

## Abbreviations

HEV: Hepatitis E virus
HEV 1-4: Hepatitis E virus genotype 1-4
MAb: Monoclonal antibody
EASL: European Association for the Study of Liver Disease
ELISA: Enzyme-linked immunosorbent assay
MFI: Mean fluorescence intensity
FFU: Focus forming unit
IgG: Immunoglobulin G
RNA: Ribonucleic acid
RT-qPCR: Quantitative real-time reverse transcription-polymerase chain reaction
ADCC: Antibody-dependent cellular cytotoxicity
ADCP: Antibody-dependent cellular phagocytosis
CDC: Complement-dependent cytotoxicity
eHEV: Quasi-enveloped Hepatitis E virus
BSL-2: Biosafety level 2
*E. coli*: Escherichia coli
SDS-PAGE: Sodium dodecyl-sulfate polyacrylamide gel electrophoresis
PBS: Phosphate buffered saline
HRP: Horseradish peroxidase
ABTS: 2,2'-Azino-bis 3-ethylbenzothiazoline−6-sulphonic acid
SDS: Sodium dodecyl sulfate
OD: Optical density
DMSO: Dimethylsulfoxide
BSA: Bovine serum albumin
TBS: Tris-buffered saline
PVDF: Polyvinylidene fluoride
RVFV: Rift valley fever virus
EMEM: Eagle’s minimal essential medium
FCS: Fetal calf serum
NEAA: Non-essential amino acids
PFA: Paraformaldehyde
HCV: Hepatitis C virus
ddPCR: Digital droplet PCR
