# Correction: Therapeutic treatment of hepatitis E virus infection in pigs with a neutralizing monoclonal antibody

**DOI:** 10.1038/s41598-025-01512-2

**Published:** 2025-05-22

**Authors:** Isabella Hrabal, Elmira Aliabadi, Sven Reiche, Saskia Weber, Cora M. Holicki, Laura Schmid, Christine Fast, Charlotte Schröder, Benjamin Gutjahr, Patrick Behrendt, Martin H. Groschup, Martin Eiden

**Affiliations:** 1 Institute for Novel and Emerging Infectious Diseases, Friedrich-Loeffler-Institut, Greifswald – Insel Riems, Germany; 2 Institute for Experimental Virology, Centre for Experimental and Clinical Infection Research, TWINCORE, Hannover, Germany; 3 Helmholz Center for Infection Research GmbH, Braunschweig, Germany; 4 Department of Experimental Animal Facilities and Biorisk Management, Friedrich-Loeffler-Institut, Greifswald – Insel Riems, Germany; 5 Institute of Diagnostic Virology, Friedrich-Loeffler-Institut, Greifswald – Insel Riems, Germany; 6 Department of Viroscience, Erasmus Medical Center, Rotterdam, The Netherlands; 7 Institute of Molecular Virology and Cell Biology, FriedrichLoeffler-Institut, Greifswald – Insel Riems, Germany; 8 Department of Gastroenterology, Hepatology, Infectious Diseases and Endocrinology, Hannover Medical School, Hannover, Germany; 9 German Centre for Infection Research, Partner Site Braunschweig-Hannover, Braunschweig, Germany; 10 German Centre for Infection Research, Partner Site Hamburg-Lübeck-Borstel-Riems, Greifswald – Insel Riems, Germany

Correction to: *Scientifc Reports* 10.1038/s41598-025-95992-x, published online 28 March 2025

The original version of this Article contained an error in Figure 2, where panel (A) did not display correctly due to an inadvertent technical issue during production. The original Figure 2 and accompanying legend appear below.

**Fig. 2:**
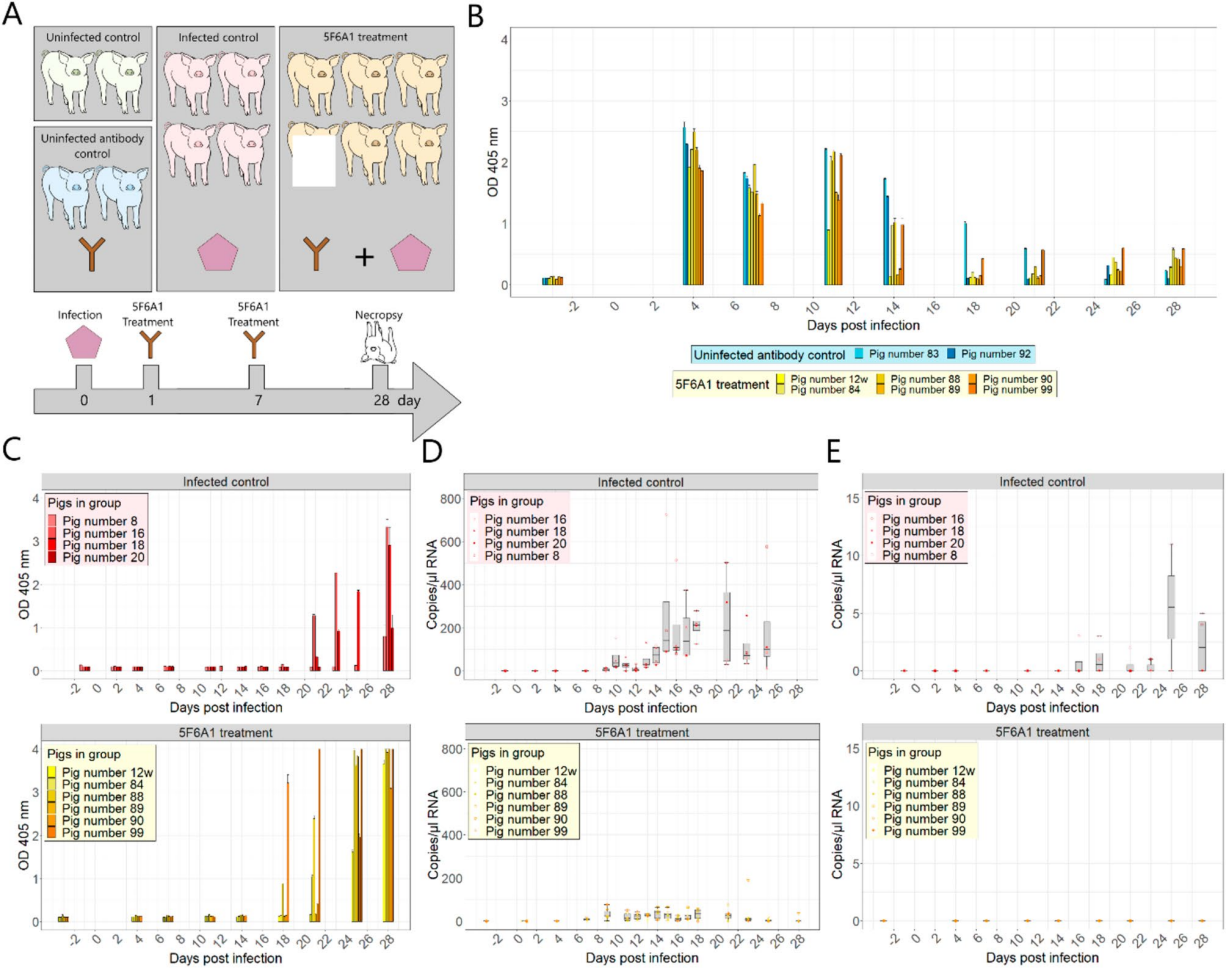
The HEV treatment experiment in pigs. (**A**) Overview of the experimental design in the 5F6A1 treatment experiment. (**B**) Partial HEV capsid protein p239 ELISA using rabbit anti mouse antibody as secondary antibody for detection of applicated murine mAb 5F6A1. Samples were run in duplicates. Depicted are the means and standard error of measurements. (**C**) Partial HEV capsid protein p239 ELISA using protein G as the secondary antibody for detection of swine antibodies after infection. Samples were run in duplicates. (**D**) HEV-3 RNA in fecal samples, detected by RT-qPCR. (**E**) HEV-3 RNA in serum samples, detected by RT-qPCR. Note the different scaling of the y-axes in comparison to “D”. Bars depict mean values. Error bars depict the standard error. Box-and-whisker plots show mean and interquartile range.

The original Article has been corrected.

