# Supplementary material for: Therapeutic treatment of hepatitis E virus infection in pigs with a neutralizing monoclonal antibody
Source: Sci Rep. 2025 Mar 28;15:10795. doi: 10.1038/s41598-025-95992-x (PMC11953370; doi:10.1038/s41598-025-95992-x)
Supplement: Supplementary file 1 — Supplementary Material 1 [file 41598_2025_95992_MOESM1_ESM.docx]

Supplementary material

## Abbreviations:

SDS-PAGE Sodium dodecyl sulfate–polyacrylamide gel electrophoresis

mab Monoclonal antibody

ELISA Enzyme-linked immunosorbent assay

HEV Hepatitis E virus

ORF Open reading frame

HEV Hepatitis E virus

RVFV Rift Valley Fever virus

DNA Desoxyribonucleic acid

*E. coli Escherichia coli*

HCV Hepatitis C virus

CS Clinical score

Dpi days post infection

## Supplemental Figure 1


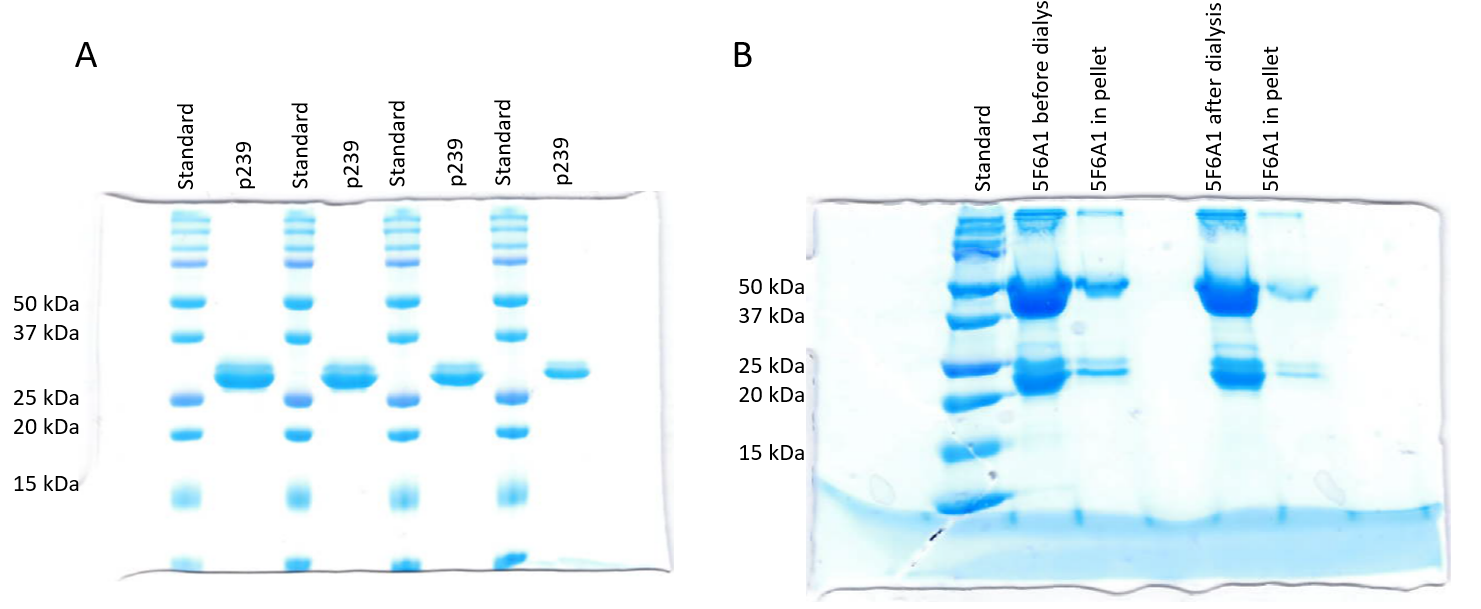


Supplemental Figure 1: Sodium dodecyl sulfate–polyacrylamide gel electrophoresis (SDS-PAGE) of p239 immunogen and applied mab.

A) SDS-PAGE of the p239 protein used for vaccinating mice. The recombinant capsid protein was expressed in E. coli BL21(DE3) (Thermo Fisher Scientific, Life Technologies GmbH, Darmstadt, Germany) and subsequently purified via Ni-NTA columns by a protocol based on established procedures (Qiagen manual, Qiagen, Hilden, Germany) under denaturating conditions (Material and Methods, 4.2, [1]) Serial dilution of the p239 immunogen starting from 0.5 mg/ml (1:1, 1:5 and 1:10) are displayed.

B) SDS-PAGE of monoclonal antibody (mab) 5F6A1 before dialysis (left) and after dialysis (right). Shown are from left to right the standard, the protein fractions in the purification medium supernatant after centrifugation, the protein fractions in the the pellet after centrifugation, the protein fractions after dialysis in PBS and centrifugation in the supernatant and the protein fractions after centrifugation of this dialysed proteins in the pellet. The 5F6A1 fraction of the supernatant post-dialysis and post centrifugation was used for pig treatment. The uncut gels were inserted for both images.

## Supplemental Figure 2


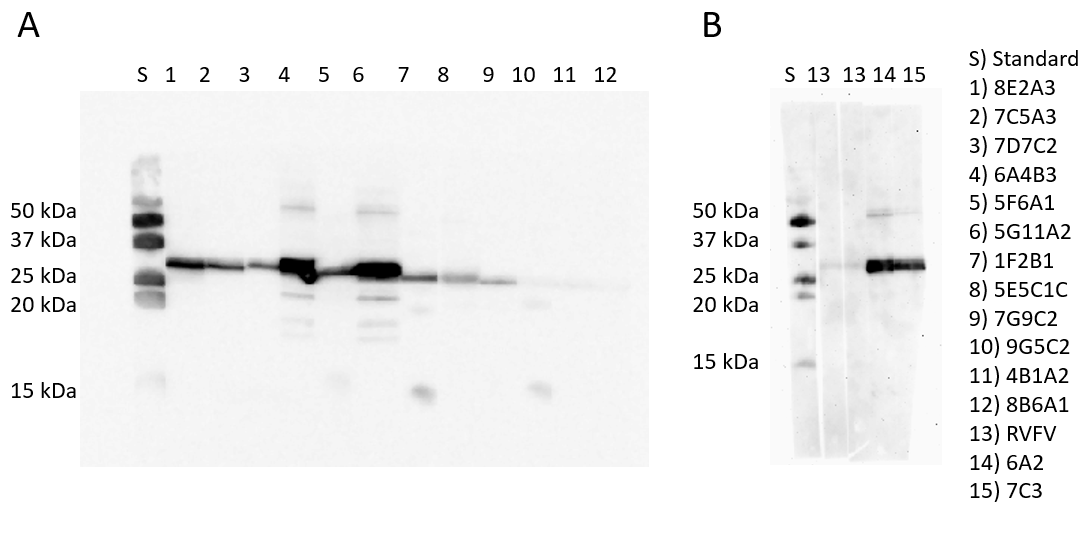
Supplemental Figure 2: Western blot of mabs against Hepatitis E virus (HEV) partial capsid protein p239 [1],) 2. A) Western blot of p239-directed antibodies blotted against partial HEV-capsid protein p239. The nine HEV-p239 - directed antibodies (1-9) are depicted followed from to three inhouse HEV-ORF 3-directed antibodies (10-12) as negative controls. B) Control Western blot. Rift Valley Fever virus (RVFV) NP9 directed antibody (13 [2]) and two inhouse ORF 2-directed antibodies (6A2 and 7C3) (14-15) in Western blot against p239 blotting protein. To evaluate the specificity of multiple mAbs simultaneously, the membrane was split, processed individually for each mab and then reassembled for scanning

## Supplemental Figure 3

**Anti-HCV mab**


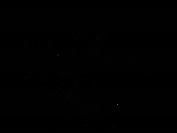


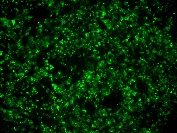

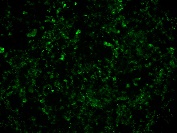

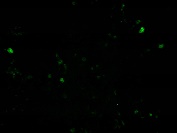

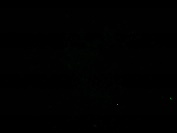

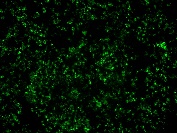

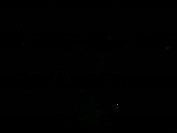

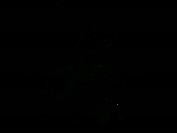

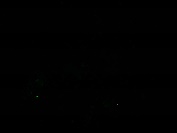

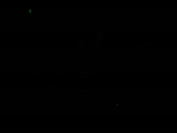

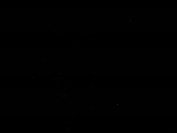

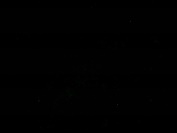

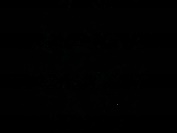

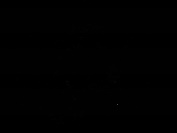

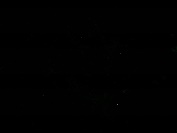

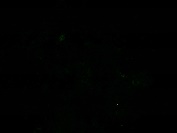

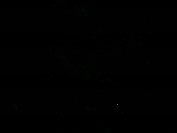

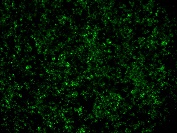

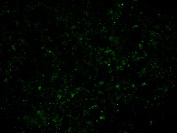

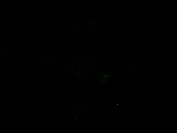

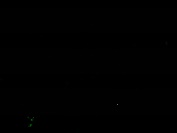

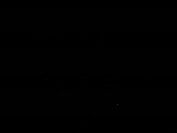

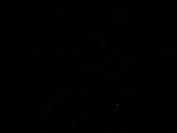

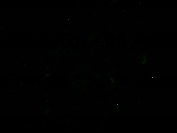

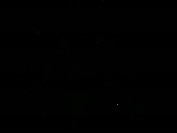

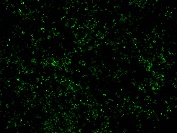

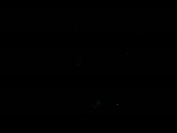

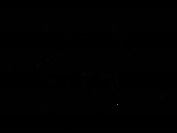

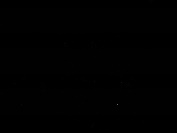


**8E2A3**

**7C5A3**

**7D7C2**

**6A4B3**

**5F6A1**

**5G11A2**

**1F2B1**

**C1/p6**

**pUC83-2**

**gt1 Sar55**

**GLUC (control)**


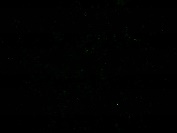

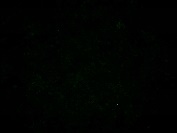

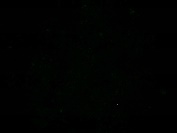

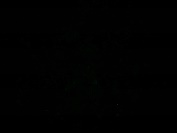

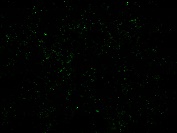

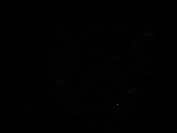

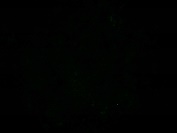

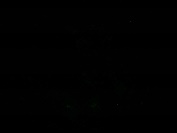


**5E5C1C**

**7G9C2**


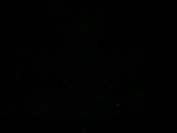


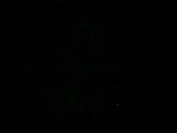


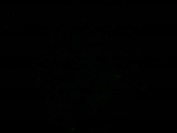


*Supplemental Figure 3: Immunofluorescence assay of mabs in human hepatocellular carcinoma HepG2/C3A cells. A Hepatitis C virus (HCV) directed antibody and glucose transfected HepG2/C3A cells were used as negative controls.* *Gaussia princeps luciferase (GLUC)*

## Supplemental Figure 4

A

| **mab** | **IC-50 (µg/ml)** |
| --- | --- |
| mab 5 (5F6A1) | 1,03E-03 |
| mab 2 (7C5A3) | 1,71E-02 |
|  | **IC-90 (µg/ml)** |
| mab 5 (5F6A1) | 0,12 |
| mab 2 (7C5A3) | 7,97 |

B C

Supplemental Figure 4: The neutralization capacity of selected mabs (5F6A1, 7C5A3 and 1F2B1) against the non-enveloped form of HEV-3 Kernow C1 clone p6 was examined using a 10-fold dilution series starting at concentrations of 5 µg/ml. All mabs exhibited a neutralization activity at a concentration of 5 µg/ml. In lower concentrations, 7C5A3, 5F6A1 demonstrated the highest neutralization capacity, indicated by the lowest number of focus forming units (FFU) per well. IC-50 and IC90-were calculated from both mab with corresponding dilution series (B) and are displayed in table C. The results demonstrated that mab 5F6A1 was the most effective antibody.

## Supplemental Figure 5


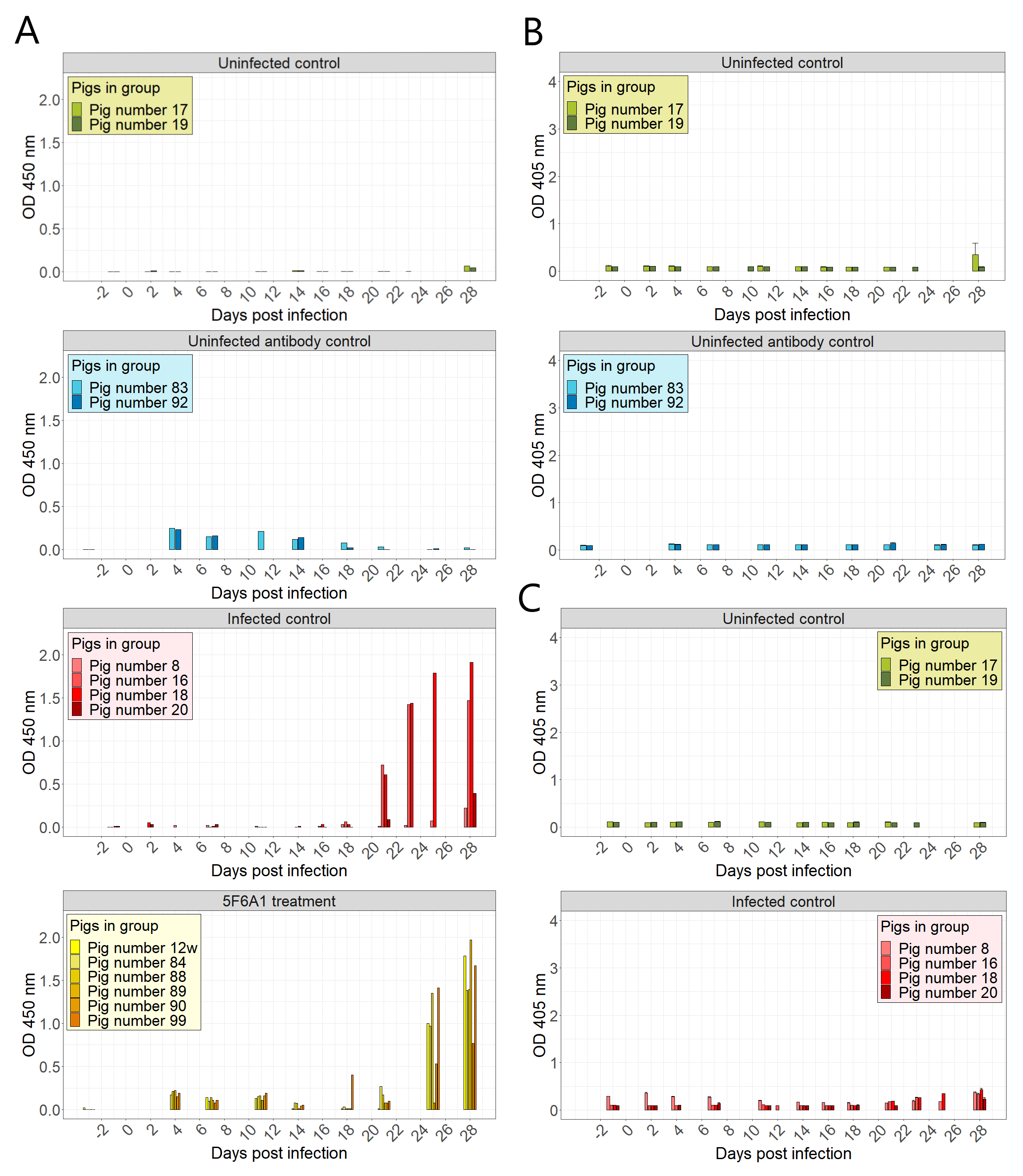


Supplemental Figure 5: A) Pig serum samples, tested in the commercially available multispecies ID-Vet ELISA. B) p239 ELISA for detection of anti-Hepatitis E virus (HEV) antibodies in uninfected groups. Displayed are the uninfected control (top) and the uninfected antibody control (bottom), that were not included into Figure 1 in the main text. C) p239 ELISA using rabbit anti-mouse antibody as secondary antibody for detection of mural mab 5F6A1. Displayed are the uninfected control (top) and the infected control (bottom), that were not displayed in Figure 1 in the main manuscript. Both were not treated with 5F6A1.

## Supplemental Table 1

Supplemental Table 1: Peptide sequences. Peptides are 20 amino acids long and have 12 overlapping amino acids. Sequences cover 239 amino acids of the Hepatitis E virus (HEV) open reading frame (ORF) 2 capsid protein.

| **Index** | **Sequence** | **Comment** |
| --- | --- | --- |
| 1 | BXVGEVGRGIALTLFNLADTLL | B=Biotin, X=Ttds |
| 2 | BXFNLADTLLGGLPTELISSAG | B=Biotin, X=Ttds |
| 3 | BXTELISSAGGQLFYSRPVVSA | B=Biotin, X=Ttds |
| 4 | BXYSRPVVSANGEPTVKLYTSV | B=Biotin, X=Ttds |
| 5 | BXTVKLYTSVENAQQDKGIAIP | B=Biotin, X=Ttds |
| 6 | BXQDKGIAIPHDIDLGDSRVVI | B=Biotin, X=Ttds |
| 7 | BXLGDSRVVIQDYDNQHEQDRP | B=Biotin, X=Ttds |
| 8 | BXNQHEQDRPTPSPAPSRPFSV | B=Biotin, X=Ttds |
| 9 | BXAPSRPFSVLRANDVLWLSLT | B=Biotin, X=Ttds |
| 10 | BXDVLWLSLTAAEYDQTTYGSS | B=Biotin, X=Ttds |
| 11 | BXDQTTYGSSTNPMYVSDTVTF | B=Biotin, X=Ttds |
| 12 | BXYVSDTVTFVNVATGAQAVAR | B=Biotin, X=Ttds |
| 13 | BXTGAQAVARSLDWSKVTLDGR | B=Biotin, X=Ttds |
| 14 | BXSKVTLDGRPLTTIQQYSKTF | B=Biotin, X=Ttds |
| 15 | BXIQQYSKTFYVLPLRGKLSFW | B=Biotin, X=Ttds |
| 16 | BXLRGKLSFWEAGTTKSGYPYN | B=Biotin, X=Ttds |
| 17 | BXTKSGYPYNYNTTASDQILIE | B=Biotin, X=Ttds |
| 18 | BXASDQILIENAAGHRVAISTY | B=Biotin, X=Ttds |
| 19 | BXHRVAISTYTTSLGAGPVSIS | B=Biotin, X=Ttds |
| 20 | BXGAGPVSISAVGVLAPHSALA | B=Biotin, X=Ttds |
|  |  |  |

Ttds; 18mer spacer

## Supplemental Table 2

*Supplemental Table 2: Blood chemistry of piglets before infection and at necropsy (28 days post infection). The measurement was conducted using the Vetscan V2 Chemistry Analyser (Abraxis) and the comprehensive diagnostic profile. Elevated enzymes are marked red, lowered enzymes are marked blue. Blood chemistry was performed with serum samples at the time point of necropsy. (ALB: Albumin, ALP: Alkaline Phosphatase, ALT: Alanine Aminotransferase, TBIL: Total Bilirubin, BUN: Blood Urea Nitrogen, Ca: Calcium, PHOS: Phosphorus, CRE: Creatinine, Glu: Clucose, Na: Sodium, K: Potassium, TP: Total Protein, Glob: Globulin.*

| Dpi | Number | Group | ALB | ALP | ALT | Amylase | TBIL | BUN | Ca | PHOS | CRE | Glu | Na | K | TP | Glob |
| --- | --- | --- | --- | --- | --- | --- | --- | --- | --- | --- | --- | --- | --- | --- | --- | --- |
| -1 | pig number 8 | infected control | 41 | 120 | 34 | 1470 | 5 | 1.5 | 2.67 | 3.75 | 111 | 4.9 | 141 | >8,5 | 62 | 22 |
| 28 | pig number 8 | infected control | 39 | 205 | 44 | 1481 | 5 | 2.7 | 2.56 | 3.45 | 131 | 5.6 | 132 | 6.6 | 63 | 25 |
| -1 | pig number 16 | infected control | 42 | 142 | 45 | 1605 | 5 | 2.1 | 2.53 | 3.42 | 134 | 4.5 | 132 | 6.4 | 56 | 14 |
| -1 | pig number 18 | infected control | 46 | 130 | 44 | 1297 | 5 | 1.5 | 2.69 | 3.86 | 123 | 5.4 | 136 | 8.2 | 62 | 16 |
| 28 | pig number 18 | infected control | 45 | 152 | 54 | 1351 | 5 | 3.7 | 2.56 | 3.23 | 136 | 4.3 | 132 | 7 | 62 | 17 |
| -1 | pig number 20 | infected control | 38 | 138 | 35 | 2302 | 5 | 1.3 | 2.48 | 3.45 | 119 | 4.8 | 138 | 7.9 | 58 | 21 |
| 28 | pig number 20 | infected control | 36 | 158 | 40 | 2353 | 5 | 3.6 | 2.32 | 3.34 | 153 | 4.9 | 129 | 7.9 | 57 | 21 |
| -1 | pig number 17 | uninfected control | 41 | 227 | 35 | 2766 | 5 | 2.4 | 2.38 | 3.63 | 141 | 4.5 | 130 | 6.2 | 55 | 14 |
| 28 | pig number 17 | uninfected control | 36 | 190 | 40 | 2560 | 5 | 3.6 | 2.51 | 3.46 | 106 | 5 | 134 | 6.7 | 56 | 19 |
| -1 | pig number 19 | uninfected control | 29 | 164 | 41 | 1836 | <2 | 1.8 | 2.52 | 3.55 | 85 | 5.1 | 135 | 8.1 | 57 | 17 |
| 28 | pig number 19 | uninfected control | 39 | 164 | 40 | 1713 | 5 | 2.3 | 2.55 | 3.36 | 102 | 5.6 | 135 | >8,5 | 59 | 19 |
| -3 | pig number 83 | Antibody treatment control | 30 | 250 | 51 | 2236 | <2 | 1.2 | 2.8 | 2.91 | 79 | 7.3 | 133 | 6.7 | 55 | 26 |
| 28 | pig number 83 | Antibody treatment control | 34 | 184 | 60 | 2419 | 5 | 2.7 | 2.4 | 3.64 | 87 | 5.2 | 131 | 6.8 | 54 | 20 |
| -3 | pig number 92 | Antibody treatment control | 40 | 255 | 50 | 1634 | 5 | 2.4 | 3.1 | 3.16 | 78 | 6.9 | 137 | 7.5 | 53 | 13 |
| 28 | pig number 92 | Antibody treatment control | 42 | 196 | 52 | 1644 | 5 | 1.3 | 2.51 | 3.49 | 93 | 5.7 | 133 | 7.2 | 59 | 17 |
| -3 | pig number 12w | Antibody treatment group | 32 | 181 | 57 | 1315 | 5 | <0,7 | 2.54 | 3.59 | 54 | 5.6 | 139 | >8,5 | 47 | 15 |
| 28 | pig number 12w | Antibody treatment group | 40 | 152 | 57 | 1312 | 5 | 2.4 | 2.54 | 3.65 | 102 | 6.3 | 139 | >8,5 | 57 | 18 |
| -3 | pig number 84 | Antibody treatment group | 39 | 152 | 67 | 2526 | 5 | 1.3 | 2.71 | 3.35 | 86 | 5.2 | 132 | 7.3 | 54 | 14 |
| 28 | pig number 84 | Antibody treatment group | 39 | 132 | 74 | 2637 | 5 | 2.2 | 2.55 | 3.67 | 102 | 6.5 | 133 | 7.1 | 58 | 20 |
| -3 | pig number 88 | Antibody treatment group | 38 | 194 | 58 | 1736 | 5 | 1.1 | 2.43 | 3.77 | 78 | 3.4 | 137 | 8.1 | 52 | 14 |
| 28 | pig number 88 | Antibody treatment group | 43 | 183 |  | 1687 | <2 | 1.7 | 2.68 | 3.82 | 109 | 5.8 | 136 | 7.7 | 61 | 18 |
| -3 | pig number 89 | Antibody treatment group | 43 | 182 | 60 | 2223 | 5 | 0.8 | 2.71 | 2.92 | 79 | 5.6 | 137 | 7.8 | 56 | 13 |
| 28 | pig number 89 | Antibody treatment group | 41 | 132 | 49 | 1894 | 5 | 1.2 | 2.63 | 3.48 | 107 | 6.2 | 137 | >8,5 | 56 | 15 |
| -3 | pig number 90 | Antibody treatment group | 39 | 220 | 43 | 1898 | 5 | 0.8 | 2.58 | 3 | 89 | - | 140 | - | 55 | 16 |
| 28 | pig number 90 | Antibody treatment group | 43 | 173 | 44 | 1909 | 5 | 1.6 | 2.67 | 3.61 | 107 | 6.3 | 135 | 7.2 | 61 | 18 |
| -3 | pig number 99 | Antibody treatment group | 36 | 203 | 42 | 1812 | 5 | 0.8 | 2.68 | 3.07 | 75 | 5 | 135 | 8 | 62 | 26 |
| 28 | pig number 99 | Antibody treatment group | 42 | 165 | 45 | 1679 | 5 | 1.9 | 2.62 | 3.53 | 107 | 6.1 | 137 | >8,5 | 60 | 18 |

## Supplemental Table 3

Supplemental Table 3: Overview of p values obtained by statistical testing. The statistical analysis of the animal test data was performed using the R statistical software (R Core Team [2023]. _R: A Language and Environment for Statistical Computing_. R Foundation for Statistical Computing, Vienna, Austria). Data are positive skewed distributed. In the context of the t-test, the null hypothesis (H0) proposed that the mean of the antibody treatment group exceeded that of the infected control group, while the alternative hypothesis (H1) suggested the contrary: H0 = µ_treatment_ ≥ µ_infected control_; H1= µ_treatment_ < µ_infected control_.

|  | Welch two-sample t-test (one-tailed) | Welch two-sample t-test (two-tailed) | Wilcoxon Rank Sum Test |
| --- | --- | --- | --- |
| HEV-RNA in serum (infected control group vs. treatment group) | p-value = 0.01305 | p-value = 0.0261 | W = 1431, p-value = 0.00132 |
| HEV-RNA in feces (infected control group vs. treatment group) | p-value = 3.685e-05 | p-value = 5.769e-05 | W = 4485, p-value = 0.0006507 |
| HEV-RNA in liver (infected control group vs. treatment group) | p-value = 0.1923 | p-value = 0.3846 | W = 21, p-value = 0.06789 |
| HEV-RNA in gallbladder (control group vs. treatment group) | p-value = 0.1955 | p-value = 0.391 | W = 15, p-value = 0.2207 |
| HEV-RNA in spleen (infected control group vs. treatment group) | p-value = 0.1014 | p-value = 0.2028 | W = 18, p-value = 0.06789 |
| HEV-RNA in bile (infected control group vs. treatment group) | p-value = 0.1898 | p-value = 0.3796 | W = 22, p-value = 0.03248 |
| HEV-RNA in feces (male vs. female in infected control group) | - | p-value = 0.2422 | W = 372.5, p-value = 0.3805 |

## Supplemental Table 4

Supplemental Table 4: Overview of clinical score (CS) sheets during the pig treatment experiment. Pig 17 in the uninfected control group was treated with non-steroidal anti-inflammatory drugs (NSAIDs) for one day, three days prior to infection, due to a painful swelling in the tarsal joint. Temperature was measured shortly after cleaning of stables.

| dpi | event | Temperature [°C]  uninfected control | | Temperature [°C]  infected control | | | | Temperature [°C]  uninfected antibody control | | Temperature [°C]  antibody treatment group | | | | | | CS liveliness | CS posture |  | CS respira-tion | CS move-ment | CS lame-ness | CS skin | CS eyes | CS faeces | CS appe-tite | CS feeding beha-viour |
| --- | --- | --- | --- | --- | --- | --- | --- | --- | --- | --- | --- | --- | --- | --- | --- | --- | --- | --- | --- | --- | --- | --- | --- | --- | --- | --- |
|  |  | Eartag nr. 17 | Eartag nr. 19 | Eartag nr. 8 | Eartag nr. 16 | Eartag nr. 18 | Eartag nr. 20 | Eartag nr. 83 | Eartag nr. 92 | Eartag nr. 12w | Eartag nr. 84 | Eartag nr. 88 | Eartag nr. 89 | Eartag nr. 90 | Eartag nr. 99 |  |  |  |  |  |  |  |  |  |  |  |
| 0 | infection |  |  |  |  |  |  | 38.9 | 39.2 |  |  |  |  |  |  | 0 | 0 |  | 0 | 0 | 0 | 0 | 0 | 0 | 0 | 0 |
| 1 | treatment |  |  |  |  |  |  | 38.7 | 39.2 | 39.2 | 39.2 | 39.2 | 39.4 | 39 | 39 | 0 | 0 |  | 0 | 0 | 0 | 0 | 0 | 0 | 0 | 0 |
| 2 |  | 38.8 | 38.1 | 38.7 | 39.1 | 38.1 | 38.6 | 39.5 | 39.7 | 39 | 39.1 | 38.8 | 39.5 | 39.2 | 39.1 | 0 | 0 |  | 0 | 0 | 0 | 0 | 0 | 0 | 0 | 0 |
| 3 |  | 38.1 | 38.3 |  |  |  |  | 39.4 | 39.4 | 39.2 | 39.1 | 39 | 38.7 | 39.1 | 39.2 | 0 | 0 |  | 0 | 0 | 0 | 0 | 0 | 0 | 0 | 0 |
| 4 |  | 38.7 | 38.6 | 39.6 | 39.5 | 39.5 | 39.5 | 39.1 | 39.4 | 39 | 39.1 | 38.6 | 39.1 | 39.2 | 38.8 | 0 | 0 |  | 0 | 0 | 0 | 0 | 0 | 0 | 0 | 0 |
| 5 |  | 38 | 38.9 | 38.8 | 38.9 | 38.8 | 39.2 | 39 | 39.1 | 39 | 39.5 | 39 | 39.2 | 39.2 | 39 | 0 | 0 |  | 0 | 0 | 0 | 0 | 0 | 0 | 0 | 0 |
| 6 |  | 38 | 38.9 | 38.9 | 39.4 | 39 | 39.2 | 39.4 | 39.2 | 39.2 | 39.2 | 38.8 | 39.1 | 39.1 | 39.1 | 0 | 0 |  | 0 | 0 | 0 | 0 | 0 | 0 | 0 | 0 |
| 7 | treatment | 39 | 39.3 | 38.5 | 38 | 38.5 | 39 | 38.8 | 39.3 | 39.4 | 39.8 | 39.1 | 38.8 | 39.2 | 39 | 0 | 0 |  | 0 | 0 | 0 | 0 | 0 | 0 | 0 | 0 |
| 8 |  | 39.3 | 39.4 | 39.5 | 39.4 | 39.4 | 39.4 | 39.3 | 39.3 | 39.2 | 39 | 38.9 | 39 | 39.1 | 38.8 | 0 | 0 |  | 0 | 0 | 0 | 0 | 0 | 0 | 0 | 0 |
| 9 |  | 39.2 | 39.2 | 38.9 | 39.2 | 38.9 | 39.1 | 39 | 39.3 | 39.4 | 39.3 | 39.1 | 39.8 | 39.7 | 39.4 | 0 | 0 |  | 0 | 0 | 0 | 0 | 0 | 1 (83),  0 (others) | 0 | 0 |
| 10 |  | 38.7 | 39.1 | 38.9 | 39.3 | 39.1 | 39.2 | 39.1 | 39.4 | 39.5 | 39.3 | 39.4 | 39.6 | 39.8 | 39.2 | 0 | 0 |  | 0 | 0 | 0 | 0 | 0 | 0 | 0 | 0 |
| 11 |  | 39.1 | 39.1 | 39.1 | 39.7 | 39.4 | 39.3 | 39.2 | 39.5 | 39.3 | 39.2 | 39.4 | 39.5 | 39.4 | 38.9 | 0 | 0 |  | 0 | 0 | 0 | 0 | 0 | 0 | 0 | 0 |
| 12 |  | 38.7 | 39.2 | 39.1 | 39.2 | 39.2 | 39.2 | 39.1 | 39.7 | 39.1 | 39.1 | 38.8 | 39 | 39.2 | 39.2 | 0 | 0 |  | 0 | 0 | 0 | 0 | 0 | 1 (83),  0 (others) | 0 | 0 |
| 13 |  | 39.2 | 39.8 | 39.5 | 40 | 39.4 | 39.5 | 39 | 39.2 | 39.1 | 38.9 | 39 | 38.6 | 39.1 | 38.6 | 0 | 0 |  | 0 | 0 | 0 | 0 | 0 | 0 | 0 | 0 |
| 14 |  |  |  | 39.7 | 39.8 | 39.4 | 39.6 | 39.6 | 39.6 | 39.4 | 39.5 | 39.2 | 38.4 | 39.1 | 39 | 0 | 0 |  | 0 | 0 | 0 | 0 | 0 | 0 | 0 | 0 |
| 15 |  |  |  | 39.5 | 39.5 | 39.1 | 39.5 | 39.6 | 39.6 | 39.4 | 39.4 | 39.4 | 39.4 | 39.5 | 39.3 | 0 | 0 |  | 0 | 0 | 0 | 0 | 0 | 0 | 0 | 0 |
| 16 |  | 39.7 | 39.8 | 39.7 | 39.4 | 39.7 | 39.6 | 39.6 | 39.2 | 39.3 | 39.2 | 38.6 | 39.4 | 39.5 | 39.4 | 0 | 0 |  | 0 | 0 | 0 | 0 | 0 | 1 (89, 90, 99),  0 (others) | 0 | 0 |
| 17 |  | 38.7 | 391 | 38.9 | 39.5 | 39.4 | 39.3 | 39.3 | 39.3 | 39.3 | 39.6 | 39.2 | 39.6 | 39.4 | 39.4 | 0 | 0 |  | 0 | 0 | 0 | 0 | 0 | 0 | 0 | 0 |
| 18 |  | 39.1 | 39.6 | 39.3 | 39.5 | 38.8 | 39.1 | 39.2 | 39.4 | 39.4 | 39.2 | 39.2 | 39.2 | 39.2 | 39.2 | 0 | 0 |  | 0 | 0 | 0 | 0 | 0 | 0 | 0 | 0 |
| 19 |  | 39 | 39.8 | 39 | 39.2 | 39 | 39.3 | 39.2 | 39.4 | 39 | 39.7 | 39.5 | 39.4 | 39.5 | 39.4 | 0 | 0 |  | 0 | 0 | 0 | 0 | 0 | 0 | 0 | 0 |
| 20 |  | 39 | 38.9 | 39.2 | 39.4 | 39.2 | 39.4 | 39.2 | 39.1 | 39.2 | 39.7 | 39.2 | 39.6 | 39.6 | 39.5 | 1 (99),  0 (others) | 1 (99),  0 (others) |  | 0 | 0 | 0 | 0 | 0 | 0 | 0 | 0 |
| 21 |  | 39 | 38.9 | 39 | 39.1 | 38.7 | 39.3 | 39.3 | 39.2 | 39.3 | 39.6 | 39.7 | 39.7 | 39.7 | 39.5 | 1 (99),  0 (others) | 1 (99),  0 (others) |  | 0 | 0 | 0 | 0 | 0 | 0 | 0 | 0 |
| 22 |  | 39.2 | 39.6 | 39.7 | 39.7 | 39.4 | 39.6 | 39.3 | 39.4 | 39.1 | 39.1 | 39.4 | 39.3 | 39.2 | 39.4 | 1 (99),  0 (others) | 1 (99),  0 (others) |  | 0 | 0 | 0 | 0 | 0 | 0 | 0 | 0 |
| 23 |  | 39.1 | 39.6 | 39.5 | 39.8 | 39.4 | 39.6 | 39.5 | 39.2 | 39.3 | 39 | 39.1 | 39.2 | 39 | 39.2 | 0 | 0 |  | 0 | 0 | 0 | 0 | 0 | 0 | 0 | 0 |
| 24 |  | 39.4 | 39.6 | 39.8 | 39.5 | 39.5 | 39.6 | 39.5 | 39.4 | 39.5 | 39.6 | 39.3 | 39.3 | 39.3 | 39.2 | 0 | 0 |  | 0 | 0 | 0 | 0 | 0 | 0 | 0 | 0 |
| 25 |  | 39.1 | 39.7 | 39.2 | 39.6 | 39.1 | 39.5 | 39.2 | 39.4 | 39.4 | 39.3 | 39.4 | 39.1 | 39.3 | 39.1 | 0 | 0 |  | 0 | 0 | 0 | 0 | 0 | 0 | 0 | 0 |
| 26 |  | 39.1 | 39.8 | 38.8 | 39.5 | 39.5 | 39.4 |  |  | 39.3 | 39.1 | 39.2 | 39.3 | 39.2 | 39.4 | 0 | 0 |  | 0 | 0 | 0 | 0 | 0 | 1 (17 and 19),  0 (others) | 0 | 0 |
| 27 |  |  |  |  |  |  |  |  |  | 39.5 | 39.2 | 39.6 | 39.1 | 39.5 | 39.3 | 0 | 0 |  | 0 | 0 | 0 | 0 | 0 | 0 | 0 | 0 |
| 28 | Necropsy |  |  |  |  |  |  |  |  | 39.1 | 39.2 | 39.1 | 39.2 | 39.1 | 39.1 | 0 | 0 |  | 0 | 0 | 0 | 0 | 0 | 0 | 0 | 0 |

## Supplemental Table 5

Supplemental Table 5: Table for statistical analysis. The table includes the following parameters: Days post-infection (dpi), experimental group assignment (group), gender (female [f] or male [m]), with columns labeled ‘gender’ and ‘group.fm’. The grouping for Wilcoxon test analysis is shown in ‘group.wilcox’. Additional columns include the stable unit where each pig was housed (stable) and the RNA viral load in feces and serum (copies/µl RNA feces, copies/µl RNA serum). Viral RNA in organ samples is presented in Table 1 of the main text.

| dpi | number | group | group. numeric | gender | group. fm | group. wilcox | stable | copies/µl RNA feces | copies/µl RNA serum |
| --- | --- | --- | --- | --- | --- | --- | --- | --- | --- |
| -1 | pig number 8 | infected control | 1 | f | 1 | 1 | Stable 156 | 0 | 0 |
| 2 | pig number 8 | infected control | 1 | f | 1 | 1 | Stable 156 | 0 | 0 |
| 4 | pig number 8 | infected control | 1 | f | 1 | 1 | Stable 156 | 0 | 0 |
| 7 | pig number 8 | infected control | 1 | f | 1 | 1 | Stable 156 | 0 | 0 |
| 9 | pig number 8 | infected control | 1 | f | 1 | 1 | Stable 156 | 11.77 |  |
| 10 | pig number 8 | infected control | 1 | f | 1 | 1 | Stable 156 | 23.41 |  |
| 11 | pig number 8 | infected control | 1 | f | 1 | 1 | Stable 156 | 25.37 | 0 |
| 12 | pig number 8 | infected control | 1 | f | 1 | 1 | Stable 156 | 9.15 |  |
| 13 | pig number 8 | infected control | 1 | f | 1 | 1 | Stable 156 | 30.02 |  |
| 14 | pig number 8 | infected control | 1 | f | 1 | 1 | Stable 156 | 41.39 | 0 |
| 15 | pig number 8 | infected control | 1 | f | 1 | 1 | Stable 156 | 186.1 |  |
| 16 | pig number 8 | infected control | 1 | f | 1 | 1 | Stable 156 | 81.22 | 0 |
| 17 | pig number 8 | infected control | 1 | f | 1 | 1 | Stable 156 | 70.61 |  |
| 18 | pig number 8 | infected control | 1 | f | 1 | 1 | Stable 156 | 216.5 | 2.75 |
| 21 | pig number 8 | infected control | 1 | f | 1 | 1 | Stable 156 | 502.2 | 0.11 |
| 23 | pig number 8 | infected control | 1 | f | 1 | 1 | Stable 156 | 84.88 | 0.558 |
| 25 | pig number 8 | infected control | 1 | f | 1 | 1 | Stable 156 | 576.2 | 10.62 |
| 28 | pig number 8 | infected control | 1 | f | 1 | 1 | Stable 156 |  | 4.06 |
| -1 | pig number 16 | infected control | 1 | m | 2 | 1 | Stable 156 | 0 | 0 |
| 2 | pig number 16 | infected control | 1 | m | 2 | 1 | Stable 156 | 0 | 0 |
| 4 | pig number 16 | infected control | 1 | m | 2 | 1 | Stable 156 | 0 | 0 |
| 7 | pig number 16 | infected control | 1 | m | 2 | 1 | Stable 156 | 0 | 0 |
| 9 | pig number 16 | infected control | 1 | m | 2 | 1 | Stable 156 | 0 |  |
| 10 | pig number 16 | infected control | 1 | m | 2 | 1 | Stable 156 | 152.8 |  |
| 11 | pig number 16 | infected control | 1 | m | 2 | 1 | Stable 156 | 24.86 | 0 |
| 12 | pig number 16 | infected control | 1 | m | 2 | 1 | Stable 156 | 31.8 |  |
| 13 | pig number 16 | infected control | 1 | m | 2 | 1 | Stable 156 | 31.97 |  |
| 14 | pig number 16 | infected control | 1 | m | 2 | 1 | Stable 156 | 119.7 | 0 |
| 15 | pig number 16 | infected control | 1 | m | 2 | 1 | Stable 156 | 725.3 |  |
| 16 | pig number 16 | infected control | 1 | m | 2 | 1 | Stable 156 | 514.3 | 3.45 |
| 17 | pig number 16 | infected control | 1 | m | 2 | 1 | Stable 156 | 375.4 |  |
| 18 | pig number 16 | infected control | 1 | m | 2 | 1 | Stable 156 | 280.3 | 1.2 |
| 21 | pig number 16 | infected control | 1 | m | 2 | 1 | Stable 156 | 51.22 | 2.41 |
| 23 | pig number 16 | infected control | 1 | m | 2 | 1 | Stable 156 | 59.64 | 0 |
| 25 | pig number 16 | infected control | 1 | m | 2 | 1 | Stable 156 | 86.47 |  |
| 28 | pig number 16 | infected control | 1 | m | 2 | 1 | Stable 156 |  | 4.54 |
| -1 | pig number 18 | infected control | 1 | f | 1 | 1 | Stable 156 | 0 | 0 |
| 2 | pig number 18 | infected control | 1 | f | 1 | 1 | Stable 156 | 0 | 0 |
| 4 | pig number 18 | infected control | 1 | f | 1 | 1 | Stable 156 | 0 | 0 |
| 7 | pig number 18 | infected control | 1 | f | 1 | 1 | Stable 156 | 0 | 0 |
| 9 | pig number 18 | infected control | 1 | f | 1 | 1 | Stable 156 | 0 |  |
| 10 | pig number 18 | infected control | 1 | f | 1 | 1 | Stable 156 | 46.89 |  |
| 11 | pig number 18 | infected control | 1 | f | 1 | 1 | Stable 156 | 63.06 | 0 |
| 12 | pig number 18 | infected control | 1 | f | 1 | 1 | Stable 156 | 0 |  |
| 13 | pig number 18 | infected control | 1 | f | 1 | 1 | Stable 156 | 130.4 |  |
| 14 | pig number 18 | infected control | 1 | f | 1 | 1 | Stable 156 | 106.4 | 0 |
| 15 | pig number 18 | infected control | 1 | f | 1 | 1 | Stable 156 | 86.95 |  |
| 16 | pig number 18 | infected control | 1 | f | 1 | 1 | Stable 156 | 115.4 | 0 |
| 17 | pig number 18 | infected control | 1 | f | 1 | 1 | Stable 156 | 202.2 |  |
| 18 | pig number 18 | infected control | 1 | f | 1 | 1 | Stable 156 | 125.1 | 0.24 |
| 21 | pig number 18 | infected control | 1 | f | 1 | 1 | Stable 156 | 29.9 | 0 |
| 23 | pig number 18 | infected control | 1 | f | 1 | 1 | Stable 156 | 34.86 | 0 |
| 25 | pig number 18 | infected control | 1 | f | 1 | 1 | Stable 156 | 11.97 | 0 |
| 28 | pig number 18 | infected control | 1 | f | 1 | 1 | Stable 156 |  | 0 |
| -1 | pig number 20 | infected control | 1 | f | 1 | 1 | Stable 156 | 0 | 0 |
| 2 | pig number 20 | infected control | 1 | f | 1 | 1 | Stable 156 | 0 | 0 |
| 4 | pig number 20 | infected control | 1 | f | 1 | 1 | Stable 156 | 0 | 0 |
| 7 | pig number 20 | infected control | 1 | f | 1 | 1 | Stable 156 | 0 | 0 |
| 9 | pig number 20 | infected control | 1 | f | 1 | 1 | Stable 156 | 13.26 |  |
| 10 | pig number 20 | infected control | 1 | f | 1 | 1 | Stable 156 | 16.39 |  |
| 11 | pig number 20 | infected control | 1 | f | 1 | 1 | Stable 156 | 0.35 | 0 |
| 12 | pig number 20 | infected control | 1 | f | 1 | 1 | Stable 156 | 0.17 |  |
| 13 | pig number 20 | infected control | 1 | f | 1 | 1 | Stable 156 | 17.26 |  |
| 14 | pig number 20 | infected control | 1 | f | 1 | 1 | Stable 156 | 28.3 | 0 |
| 15 | pig number 20 | infected control | 1 | f | 1 | 1 | Stable 156 | 92.62 |  |
| 16 | pig number 20 | infected control | 1 | f | 1 | 1 | Stable 156 | 102.4 | 0 |
| 17 | pig number 20 | infected control | 1 | f | 1 | 1 | Stable 156 | 73.32 |  |
| 18 | pig number 20 | infected control | 1 | f | 1 | 1 | Stable 156 | 208.9 | 0.04 |
| 21 | pig number 20 | infected control | 1 | f | 1 | 1 | Stable 156 | 318.7 | 0 |
| 23 | pig number 20 | infected control | 1 | f | 1 | 1 | Stable 156 | 257.2 |  |
| 25 | pig number 20 | infected control | 1 | f | 1 | 1 | Stable 156 | 111.4 |  |
| 28 | pig number 20 | infected control | 1 | f | 1 | 1 | Stable 156 |  | 0 |
| -1 | pig number 17 | uninfected control | 4 | m |  |  | Stable 157 | 0 | 0 |
| 2 | pig number 17 | uninfected control | 4 | m |  |  | Stable 157 | 0 | 0 |
| 4 | pig number 17 | uninfected control | 4 | m |  |  | Stable 157 | 0 | 0 |
| 7 | pig number 17 | uninfected control | 4 | m |  |  | Stable 157 | 0 | 0 |
| 9 | pig number 17 | uninfected control | 4 | m |  |  | Stable 157 | 0 |  |
| 10 | pig number 17 | uninfected control | 4 | m |  |  | Stable 157 | 0 |  |
| 11 | pig number 17 | uninfected control | 4 | m |  |  | Stable 157 | 0 | 0 |
| 12 | pig number 17 | uninfected control | 4 | m |  |  | Stable 157 | 0 |  |
| 13 | pig number 17 | uninfected control | 4 | m |  |  | Stable 157 | 0 |  |
| 14 | pig number 17 | uninfected control | 4 | m |  |  | Stable 157 | 0 | 0 |
| 15 | pig number 17 | uninfected control | 4 | m |  |  | Stable 157 | 0 |  |
| 16 | pig number 17 | uninfected control | 4 | m |  |  | Stable 157 | 0 | 0 |
| 17 | pig number 17 | uninfected control | 4 | m |  |  | Stable 157 | 0 |  |
| 18 | pig number 17 | uninfected control | 4 | m |  |  | Stable 157 | 0 | 0 |
| 21 | pig number 17 | uninfected control | 4 | m |  |  | Stable 157 | 0 | 0 |
| 23 | pig number 17 | uninfected control | 4 | m |  |  | Stable 157 | 0 |  |
| 25 | pig number 17 | uninfected control | 4 | m |  |  | Stable 157 | 0 |  |
| 28 | pig number 17 | uninfected control | 4 | m |  |  | Stable 157 |  | 0 |
| -1 | pig number 19 | uninfected control | 4 | f |  |  | Stable 157 | 0 | 0 |
| 2 | pig number 19 | uninfected control | 4 | f |  |  | Stable 157 | 0 | 0 |
| 4 | pig number 19 | uninfected control | 4 | f |  |  | Stable 157 | 0 | 0 |
| 7 | pig number 19 | uninfected control | 4 | f |  |  | Stable 157 | 0 | 0 |
| 9 | pig number 19 | uninfected control | 4 | f |  |  | Stable 157 | 0 |  |
| 10 | pig number 19 | uninfected control | 4 | f |  |  | Stable 157 | 0 |  |
| 11 | pig number 19 | uninfected control | 4 | f |  |  | Stable 157 | 0 | 0 |
| 12 | pig number 19 | uninfected control | 4 | f |  |  | Stable 157 | 0 |  |
| 13 | pig number 19 | uninfected control | 4 | f |  |  | Stable 157 | 0 |  |
| 14 | pig number 19 | uninfected control | 4 | f |  |  | Stable 157 | 0 | 0 |
| 16 | pig number 19 | uninfected control | 4 | f |  |  | Stable 157 | 0 | 0 |
| 17 | pig number 19 | uninfected control | 4 | f |  |  | Stable 157 | 0 |  |
| 18 | pig number 19 | uninfected control | 4 | f |  |  | Stable 157 | 0 | 0 |
| 21 | pig number 19 | uninfected control | 4 | f |  |  | Stable 157 | 0 | 0 |
| 23 | pig number 19 | uninfected control | 4 | f |  |  | Stable 157 | 0 |  |
| 25 | pig number 19 | uninfected control | 4 | f |  |  | Stable 157 | 0 |  |
| 28 | pig number 19 | uninfected control | 4 | f |  |  | Stable 157 |  | 0 |
| -3 | pig number 83 | uninfected antibody control | 3 | m |  |  | Stable 159 | 0 | 0 |
| 1 | pig number 83 | uninfected antibody control | 3 | m |  |  | Stable 159 | 0 |  |
| 4 | pig number 83 | uninfected antibody control | 3 | m |  |  | Stable 159 | 0 | 0 |
| 7 | pig number 83 | uninfected antibody control | 3 | m |  |  | Stable 159 | 0 | 0 |
| 9 | pig number 83 | uninfected antibody control | 3 | m |  |  | Stable 159 | 0 |  |
| 11 | pig number 83 | uninfected antibody control | 3 | m |  |  | Stable 159 | 0 | 0 |
| 12 | pig number 83 | uninfected antibody control | 3 | m |  |  | Stable 159 | 0 |  |
| 13 | pig number 83 | uninfected antibody control | 3 | m |  |  | Stable 159 | 0 |  |
| 14 | pig number 83 | uninfected antibody control | 3 | m |  |  | Stable 159 | 0 | 0 |
| 15 | pig number 83 | uninfected antibody control | 3 | m |  |  | Stable 159 | 0 |  |
| 16 | pig number 83 | uninfected antibody control | 3 | m |  |  | Stable 159 | 0 |  |
| 17 | pig number 83 | uninfected antibody control | 3 | m |  |  | Stable 159 | 0 |  |
| 18 | pig number 83 | uninfected antibody control | 3 | m |  |  | Stable 159 | 0 | 0 |
| 21 | pig number 83 | uninfected antibody control | 3 | m |  |  | Stable 159 | 0 | 0 |
| 23 | pig number 83 | uninfected antibody control | 3 | m |  |  | Stable 159 | 0 |  |
| 25 | pig number 83 | uninfected antibody control | 3 | m |  |  | Stable 159 | 0 | 0 |
| 28 | pig number 83 | uninfected antibody control | 3 | m |  |  | Stable 159 | 0 | 0 |
| -3 | pig number 92 | uninfected antibody control | 3 | m |  |  | Stable 159 | 0 | 0 |
| 1 | pig number 92 | uninfected antibody control | 3 | m |  |  | Stable 159 | 0 |  |
| 4 | pig number 92 | uninfected antibody control | 3 | m |  |  | Stable 159 | 0 | 0 |
| 7 | pig number 92 | uninfected antibody control | 3 | m |  |  | Stable 159 | 0 | 0 |
| 9 | pig number 92 | uninfected antibody control | 3 | m |  |  | Stable 159 | 0 |  |
| 11 | pig number 92 | uninfected antibody control | 3 | m |  |  | Stable 159 | 0 | 0 |
| 12 | pig number 92 | uninfected antibody control | 3 | m |  |  | Stable 159 | 0 |  |
| 13 | pig number 92 | uninfected antibody control | 3 | m |  |  | Stable 159 | 0 |  |
| 14 | pig number 92 | uninfected antibody control | 3 | m |  |  | Stable 159 | 0 | 0 |
| 15 | pig number 92 | uninfected antibody control | 3 | m |  |  | Stable 159 | 0 |  |
| 16 | pig number 92 | uninfected antibody control | 3 | m |  |  | Stable 159 | 0 |  |
| 17 | pig number 92 | uninfected antibody control | 3 | m |  |  | Stable 159 | 0 |  |
| 18 | pig number 92 | uninfected antibody control | 3 | m |  |  | Stable 159 | 0 | 0 |
| 21 | pig number 92 | uninfected antibody control | 3 | m |  |  | Stable 159 | 0 | 0 |
| 23 | pig number 92 | uninfected antibody control | 3 | m |  |  | Stable 159 | 0 |  |
| 25 | pig number 92 | uninfected antibody control | 3 | m |  |  | Stable 159 | 0 | 0 |
| 28 | pig number 92 | uninfected antibody control | 3 | m |  |  | Stable 159 | 0 | 0 |
| -3 | pig number 12w | 5F6A1 treatment | 2 | m |  | 2 | Stable 153 | 0 | 0 |
| 1 | pig number 12w | 5F6A1 treatment | 2 | m |  | 2 | Stable 153 | 0 |  |
| 4 | pig number 12w | 5F6A1 treatment | 2 | m |  | 2 | Stable 153 | 0.58 | 0 |
| 7 | pig number 12w | 5F6A1 treatment | 2 | m |  | 2 | Stable 153 | 0.81 | 0 |
| 9 | pig number 12w | 5F6A1 treatment | 2 | m |  | 2 | Stable 153 | 2.1 |  |
| 11 | pig number 12w | 5F6A1 treatment | 2 | m |  | 2 | Stable 153 | 1.59 | 0 |
| 12 | pig number 12w | 5F6A1 treatment | 2 | m |  | 2 | Stable 153 | 3.74 |  |
| 13 | pig number 12w | 5F6A1 treatment | 2 | m |  | 2 | Stable 153 | 4.63 |  |
| 14 | pig number 12w | 5F6A1 treatment | 2 | m |  | 2 | Stable 153 | 7.09 | 0 |
| 15 | pig number 12w | 5F6A1 treatment | 2 | m |  | 2 | Stable 153 | 6.26 |  |
| 16 | pig number 12w | 5F6A1 treatment | 2 | m |  | 2 | Stable 153 | 1.71 |  |
| 17 | pig number 12w | 5F6A1 treatment | 2 | m |  | 2 | Stable 153 | 8.71 |  |
| 18 | pig number 12w | 5F6A1 treatment | 2 | m |  | 2 | Stable 153 | 25.07 | 0 |
| 21 | pig number 12w | 5F6A1 treatment | 2 | m |  | 2 | Stable 153 | 11.86 | 0 |
| 23 | pig number 12w | 5F6A1 treatment | 2 | m |  | 2 | Stable 153 | 14.29 |  |
| 25 | pig number 12w | 5F6A1 treatment | 2 | m |  | 2 | Stable 153 | 5.65 | 0 |
| 28 | pig number 12w | 5F6A1 treatment | 2 | m |  | 2 | Stable 153 | 0 | 0 |
| -3 | pig number 84 | 5F6A1 treatment | 2 | m |  | 2 | Stable 153 | 0 | 0 |
| 1 | pig number 84 | 5F6A1 treatment | 2 | m |  | 2 | Stable 153 | 0 |  |
| 4 | pig number 84 | 5F6A1 treatment | 2 | m |  | 2 | Stable 153 | 0.73 | 0 |
| 7 | pig number 84 | 5F6A1 treatment | 2 | m |  | 2 | Stable 153 | 3.8 | 0 |
| 9 | pig number 84 | 5F6A1 treatment | 2 | m |  | 2 | Stable 153 | 24.59 |  |
| 11 | pig number 84 | 5F6A1 treatment | 2 | m |  | 2 | Stable 153 | 16.6 | 0 |
| 12 | pig number 84 | 5F6A1 treatment | 2 | m |  | 2 | Stable 153 | 27.92 |  |
| 13 | pig number 84 | 5F6A1 treatment | 2 | m |  | 2 | Stable 153 | 26.4 |  |
| 14 | pig number 84 | 5F6A1 treatment | 2 | m |  | 2 | Stable 153 | 51.45 | 0 |
| 15 | pig number 84 | 5F6A1 treatment | 2 | m |  | 2 | Stable 153 | 30.55 |  |
| 16 | pig number 84 | 5F6A1 treatment | 2 | m |  | 2 | Stable 153 | 18.09 |  |
| 17 | pig number 84 | 5F6A1 treatment | 2 | m |  | 2 | Stable 153 | 26.61 |  |
| 18 | pig number 84 | 5F6A1 treatment | 2 | m |  | 2 | Stable 153 | 58.06 | 0 |
| 21 | pig number 84 | 5F6A1 treatment | 2 | m |  | 2 | Stable 153 | 27.82 | 0 |
| 23 | pig number 84 | 5F6A1 treatment | 2 | m |  | 2 | Stable 153 | 10.12 |  |
| 25 | pig number 84 | 5F6A1 treatment | 2 | m |  | 2 | Stable 153 | 1.43 | 0 |
| 28 | pig number 84 | 5F6A1 treatment | 2 | m |  | 2 | Stable 153 | 0 | 0 |
| -3 | pig number 88 | 5F6A1 treatment | 2 | m |  | 2 | Stable 153 | 0 | 0 |
| 1 | pig number 88 | 5F6A1 treatment | 2 | m |  | 2 | Stable 153 | 0 |  |
| 4 | pig number 88 | 5F6A1 treatment | 2 | m |  | 2 | Stable 153 | 0 | 0 |
| 7 | pig number 88 | 5F6A1 treatment | 2 | m |  | 2 | Stable 153 | 6.09 | 0 |
| 9 | pig number 88 | 5F6A1 treatment | 2 | m |  | 2 | Stable 153 | 21.46 |  |
| 11 | pig number 88 | 5F6A1 treatment | 2 | m |  | 2 | Stable 153 | 4.99 | 0 |
| 12 | pig number 88 | 5F6A1 treatment | 2 | m |  | 2 | Stable 153 | 7.16 |  |
| 13 | pig number 88 | 5F6A1 treatment | 2 | m |  | 2 | Stable 153 | 34.35 |  |
| 14 | pig number 88 | 5F6A1 treatment | 2 | m |  | 2 | Stable 153 | 36.26 | 0 |
| 15 | pig number 88 | 5F6A1 treatment | 2 | m |  | 2 | Stable 153 | 25.75 |  |
| 16 | pig number 88 | 5F6A1 treatment | 2 | m |  | 2 | Stable 153 | 15.78 |  |
| 17 | pig number 88 | 5F6A1 treatment | 2 | m |  | 2 | Stable 153 | 10.73 |  |
| 18 | pig number 88 | 5F6A1 treatment | 2 | m |  | 2 | Stable 153 | 10.64 | 0 |
| 21 | pig number 88 | 5F6A1 treatment | 2 | m |  | 2 | Stable 153 | 0.97 | 0 |
| 23 | pig number 88 | 5F6A1 treatment | 2 | m |  | 2 | Stable 153 | 1.56 |  |
| 25 | pig number 88 | 5F6A1 treatment | 2 | m |  | 2 | Stable 153 | 0 | 0 |
| 28 | pig number 88 | 5F6A1 treatment | 2 | m |  | 2 | Stable 153 | 0 | 0 |
| -3 | pig number 89 | 5F6A1 treatment | 2 | m |  | 2 | Stable 151 | 0 | 0 |
| 1 | pig number 89 | 5F6A1 treatment | 2 | m |  | 2 | Stable 151 | 0 |  |
| 4 | pig number 89 | 5F6A1 treatment | 2 | m |  | 2 | Stable 151 | 0 | 0 |
| 7 | pig number 89 | 5F6A1 treatment | 2 | m |  | 2 | Stable 151 | 10.2 | 0 |
| 9 | pig number 89 | 5F6A1 treatment | 2 | m |  | 2 | Stable 151 | 20.53 |  |
| 11 | pig number 89 | 5F6A1 treatment | 2 | m |  | 2 | Stable 151 | 39.25 | 0 |
| 12 | pig number 89 | 5F6A1 treatment | 2 | m |  | 2 | Stable 151 | 20.14 |  |
| 13 | pig number 89 | 5F6A1 treatment | 2 | m |  | 2 | Stable 151 | 29.39 |  |
| 14 | pig number 89 | 5F6A1 treatment | 2 | m |  | 2 | Stable 151 | 43.83 | 0 |
| 15 | pig number 89 | 5F6A1 treatment | 2 | m |  | 2 | Stable 151 | 38.9 |  |
| 16 | pig number 89 | 5F6A1 treatment | 2 | m |  | 2 | Stable 151 | 26.1 |  |
| 17 | pig number 89 | 5F6A1 treatment | 2 | m |  | 2 | Stable 151 | 64.64 |  |
| 18 | pig number 89 | 5F6A1 treatment | 2 | m |  | 2 | Stable 151 | 45.54 | 0 |
| 21 | pig number 89 | 5F6A1 treatment | 2 | m |  | 2 | Stable 151 | 76.84 | 0 |
| 23 | pig number 89 | 5F6A1 treatment | 2 | m |  | 2 | Stable 151 | 11.63 |  |
| 25 | pig number 89 | 5F6A1 treatment | 2 | m |  | 2 | Stable 151 | 1.31 | 0 |
| 28 | pig number 89 | 5F6A1 treatment | 2 | m |  | 2 | Stable 151 | 0 | 0 |
| -3 | pig number 90 | 5F6A1 treatment | 2 | m |  | 2 | Stable 151 | 0 | 0 |
| 1 | pig number 90 | 5F6A1 treatment | 2 | m |  | 2 | Stable 151 | 0 |  |
| 4 | pig number 90 | 5F6A1 treatment | 2 | m |  | 2 | Stable 151 | 0 | 0 |
| 7 | pig number 90 | 5F6A1 treatment | 2 | m |  | 2 | Stable 151 | 17.49 | 0 |
| 9 | pig number 90 | 5F6A1 treatment | 2 | m |  | 2 | Stable 151 | 52.16 |  |
| 11 | pig number 90 | 5F6A1 treatment | 2 | m |  | 2 | Stable 151 | 10.34 | 0 |
| 12 | pig number 90 | 5F6A1 treatment | 2 | m |  | 2 | Stable 151 | 31.96 |  |
| 13 | pig number 90 | 5F6A1 treatment | 2 | m |  | 2 | Stable 151 | 32.44 |  |
| 14 | pig number 90 | 5F6A1 treatment | 2 | m |  | 2 | Stable 151 | 65.38 | 0 |
| 15 | pig number 90 | 5F6A1 treatment | 2 | m |  | 2 | Stable 151 | 64.02 |  |
| 16 | pig number 90 | 5F6A1 treatment | 2 | m |  | 2 | Stable 151 | 5.33 |  |
| 18 | pig number 90 | 5F6A1 treatment | 2 | m |  | 2 | Stable 151 | 55.34 | 0 |
| 21 | pig number 90 | 5F6A1 treatment | 2 | m |  | 2 | Stable 151 | 26.39 | 0 |
| 23 | pig number 90 | 5F6A1 treatment | 2 | m |  | 2 | Stable 151 | 188.3 |  |
| 25 | pig number 90 | 5F6A1 treatment | 2 | m |  | 2 | Stable 151 | 16.69 | 0 |
| 28 | pig number 90 | 5F6A1 treatment | 2 | m |  | 2 | Stable 151 | 35.66 | 0 |
| -3 | pig number 99 | 5F6A1 treatment | 2 | m |  | 2 | Stable 151 | 0 | 0 |
| 1 | pig number 99 | 5F6A1 treatment | 2 | m |  | 2 | Stable 151 | 0 |  |
| 4 | pig number 99 | 5F6A1 treatment | 2 | m |  | 2 | Stable 151 | 1.24 | 0 |
| 7 | pig number 99 | 5F6A1 treatment | 2 | m |  | 2 | Stable 151 | 12.44 | 0 |
| 9 | pig number 99 | 5F6A1 treatment | 2 | m |  | 2 | Stable 151 | 76.89 |  |
| 11 | pig number 99 | 5F6A1 treatment | 2 | m |  | 2 | Stable 151 | 52.16 | 0 |
| 12 | pig number 99 | 5F6A1 treatment | 2 | m |  | 2 | Stable 151 | 42.65 |  |
| 13 | pig number 99 | 5F6A1 treatment | 2 | m |  | 2 | Stable 151 | 23.27 |  |
| 14 | pig number 99 | 5F6A1 treatment | 2 | m |  | 2 | Stable 151 | 5.84 | 0 |
| 15 | pig number 99 | 5F6A1 treatment | 2 | m |  | 2 | Stable 151 | 3.89 |  |
| 16 | pig number 99 | 5F6A1 treatment | 2 | m |  | 2 | Stable 151 | 1.51 |  |
| 17 | pig number 99 | 5F6A1 treatment | 2 | m |  | 2 | Stable 151 | 10.4 |  |
| 18 | pig number 99 | 5F6A1 treatment | 2 | m |  | 2 | Stable 151 | 0 | 0 |
| 21 | pig number 99 | 5F6A1 treatment | 2 | m |  | 2 | Stable 151 | 37.11 | 0 |
| 23 | pig number 99 | 5F6A1 treatment | 2 | m |  | 2 | Stable 151 | 2.36 |  |
| 25 | pig number 99 | 5F6A1 treatment | 2 | m |  | 2 | Stable 151 | 0 | 0 |
| 28 | pig number 99 | 5F6A1 treatment | 2 | m |  | 2 | Stable 151 | 0 | 0 |

## Supplemental Data 1

p429 DNA sequence (1302 nucleotides, 46.9 kDa)

CAT ATG GAG GCG AGC AAC TAT GCG CAA TAT CGG GTT GTT CGC GCA ACC ATC CGC TAT CGT CCT CTG GTT CCG AAT GCG GTC GGC GGC TAT GCG ATC TCC ATT AGC TTT TGG CCG CAG ACG ACT ACA ACC CCT ACG TCG GTC GAT ATG AAC TCT ATC ACC AGC ACA GAC GTA CGC ATT CTC GTA CAA CCG GGT ATT GCC AGC GAA CTG GTC ATT CCC AGC GAA CGT TTA CAC TAC CGC AAC CAA GGC TGG CGT TCC GTG GAG ACT AGT GGC GTA GCT GAG GAA GAA GCC ACG AGT GGG TTG GTC ATG CTG TGC ATC CAT GGG TCA CCA GTC AAC TCG TAC ACG AAT ACG CCG TAT ACT GGC GCT TTA GGC CTC CTT GAC TTT GCG CTG GAG CTC GAA TTT CGC AAT CTT ACG CCA GGC AAT ACC AAT ACC CGC GTT AGT CGC TAC ACC TCG ACA GCA CGT CAT CGT TTA CGC CGT GGT ACC GAT GGA ACC GCG GAA CTG ACT ACC ACA GCC GCA ACT CGG TTC ATG AAG GAT CTG CAC TTT ACC GGG ACC AAT GGC GTG GGC GAA GTT GGG CGC GGT ATT GCG CTG ACG CTG TTC AAT CTG GCG GAT ACG CTG CTT GGT GGC TTA CCG ACG GAA CTG ATT TCC TCT GCC GGT GGT CAG CTC TTC TAC AGC CGC CCT GTG GTA TCC GCC AAC GGA GAA CCC ACC GTG AAA TTG TAC ACG AGC GTG GAG AAT GCC CAG CAA GAT AAA GGG ATT GCG ATC CCG CAT GAC ATC GAT TTG GGT GAC TCA CGC GTT GTG ATT CAG GAT TAT GAC AAC CAG CAT GAA CAG GAT CGC CCG ACC CCG TCA CCA GCC CCG AGT CGT CCG TTT TCG GTG TTA CGC GCG AAT GAT GTC TTG TGG CTG TCA CTG ACC GCA GCT GAA TAC GAC CAG ACT ACC TAT GGC AGC TCT ACA AAC CCG ATG TAC GTT AGC GAT ACC GTG ACT TTC GTG AAC GTA GCC ACA GGT GCC CAA GCT GTT GCG CGT TCG CTG GAT TGG AGC AAA GTG ACG CTG GAT GGA CGT CCA CTG ACC ACC ATT CAG CAG TAT AGT AAG ACC TTC TAT GTG CTG CCG CTG CGC GGA AAA CTG TCC TTT TGG GAA GCG GGT ACT ACG AAA AGT GGC TAT CCC TAC AAC TAC AAC ACA ACG GCA TCT GAC CAG ATT TTG ATC GAG AAC GCC GCA GGC CAC CGT GTG GCA ATC TCT ACC TAT ACC ACC TCG CTG GGT GCA GGC CCT GTG AGC ATT TCC GCG GTT GGT GTC CTT GCT CCG CAT TCA GCT TAA CTC GAG

The sequence codes for a partial HEV capsid protein. It was codon optimized for bacterial expression in *E. coli* and synthesized by Eurofins (Eurofins Genomics Europe Shared Services GmbH, Ebersberg, Germany) and cloned via 5’-Ndel and 3’-Xhol restriction sites into the vector pET19b, that harbors a N-terminal His-tag.

pVAX1-HEV-SMP DNA sequence (1448 nucleotides, 120.76 kDa)

GGT ACC GGG GCA ATT CTC CGG AGA CAG TAC AAC CTG AGC ACA AGC CCA CTG ACA TCT AGC GTG GCT TCC GGG ACC AAC CTT GTG CTG TAT GCG GCT CCA CTG AAT CCC CTT CTG CCG CTG CAG GAT GGC ACA AAC ACT CAC ATC ATG GCA ACG GAA GCC AGC AAT TAC GCC CAG TAC AGA GTG GTC CGA GCA ACA ATC AGG TAT AGA CCC TTG GTG CCG AAT GCT GTG GGC GGG TAT GCC ATC AGC ATC TCA TTT TGG CCC CAG ACT ACC ACC ACG CCT ACT TCC GTC GAC ATG AAC AGC ATA ACT AGC ACC GAT GTA CGC ATA CTT GTA CAG CCT GGC ATT GCC TCC GAG CTC GTC ATT CCC TCT GAA CGG CTT CAC TAT CGC AAT CAA GGC TGG CGA TCT GTG GAG ACA TCA GGG GTT GCA GAG GAA GAA GCC ACG TCT GGG CTG GTG ATG CTG TGC ATT CAC GGC TCA CCC GTG AAC AGC TAC ACT AAT ACC CCT TAT ACA GGT GCC CTT GGA CTG CTG GAT TTT GCC CTG GAA CTC GAG TTT CGG AAT TTG ACA CCC GGT AAC ACC AAC ACT AGA GTG AGC CGG TAT ACT TCC ACC GCC AGA CAT CGG CTG CGA CGC GGC ACC GAT GGC ACT GCT GAG CTG ACT ACT ACG GCC GCA ACG AGG TTC ATG AAG GAC CTC CAT TTC ACC GGC ACC AAT GGT GTC GGC GAG GTT GGA CGT GGG ATA GCG CTG ACT CTG TTC AAT CTG GCC GAT ACG TTG CTC GGT GGA TTG CCC ACA GAG CTG ATC TCA TCT GCC GGA GGT CAG CTG TTC TAT TCC CGC CCT GTA GTG TCA GCC AAT GGA GAA CCA ACC GTG AAA CTG TAC ACA TCC GTG GAA AAC GCC CAG CAA GAC AAA GGA ATT GCG ATT CCT CAC GAC ATC GAC CTC GGG GAT TCC AGG GTT GTC ATC CAG GAC TAT GAC AAC CAA CAC GAG CAA GAT AGG CCA ACA CCA TCA CCA GCT CCT AGT CGG CCC TTT AGT GTG CTG AGA GCT AAT GAC GTT CTC TGG CTG TCT CTG ACA GCA GCG GAA TAC GAC CAG ACT ACC TAT GGC AGC TCC ACT AAC CCA ATG TAT GTG AGT GAT ACC GTC ACC TTC GTT AAT GTA GCC ACT GGA GCT CAA GCA GTC GCA AGG TCC CTG GAT TGG TCT AAG GTG ACA CTT GAC GGC AGG CCT TTG ACC ACC ATC CAG CAG TAC AGC AAG ACC TTC TAC GTG CTC CCT TTG CGT GGG AAA CTG AGC TTT TGG GAG GCT GGA ACC ACC AAG AGT GGC TAC CCG TAC AAC TAC AAC ACA ACA GCC AGT GAC CAG ATT CTC ATC GAG AAC GCT GCA GGA CAT CGC GTT GCC ATC TCA ACC TAC ACA ACA AGC CTG GGT GCT GGG CCA GTC TCC ATT AGT GCC GTT GGC GTG CTG GCT CCC CAT TCT TGA GCG GCC GC

The sequence was codon optimized for *homo sapiens* as a representant of mammals and synthesized by Eurofins (Eurofins Genomics Europe Shared Services GmbH, Ebersberg, Germany). The sequences codes for the three domains of the HEV capsid Protein (S, M and P) and were cloned into a modified pVAX1-Ub universal fusion vector that was kindly provided by Friedmann Weber (Institute for Virology, Justus-Liebig-University, Giessen, Germany). The vector enables expression of a 5'-ubiquitin antigen fusion protein.

## Supplemental Data 2

Supplemental Data 2: Assessment of clinical parameters and taken samples of pigs during the experiment. A clinical score over 15 and signs of acute hepatitis would have led to euthanasia.

| date/dpi | Animal number | Sampling | | Temperature | Livelyness | | | | Posture | | | | Respiration | | | | Movement | | | | Lameness | | | | Skin | | | | Eyes | | | | Faeces | | | | Appetite | | | | Feeding behaviour | | | |
| --- | --- | --- | --- | --- | --- | --- | --- | --- | --- | --- | --- | --- | --- | --- | --- | --- | --- | --- | --- | --- | --- | --- | --- | --- | --- | --- | --- | --- | --- | --- | --- | --- | --- | --- | --- | --- | --- | --- | --- | --- | --- | --- | --- | --- |
|  |  | Fecal sample | Blood |  | 0 | 1 | 2 | 3 | 0 | 1 | 2 | 3 | 0 | 1 | 2 | 3 | 0 | 1 | 2 | 3 | 0 | 1 | 2 | 3 | 0 | 1 | 2 | 3 | 0 | 1 | 2 | 3 | 0 | 1 | 2 | 3 | 0 | 1 | 2 | 3 | 0 | 1 | 2 | 3 |
|  |  |  |  |  |  |  |  |  |  |  |  |  |  |  |  |  |  |  |  |  |  |  |  |  |  |  |  |  |  |  |  |  |  |  |  |  |  |  |  |  |  |  |  |  |
|  |  |  |  |  |  |  |  |  |  |  |  |  |  |  |  |  |  |  |  |  |  |  |  |  |  |  |  |  |  |  |  |  |  |  |  |  |  |  |  |  |  |  |  |  |
|  |  |  |  |  |  |  |  |  |  |  |  |  |  |  |  |  |  |  |  |  |  |  |  |  |  |  |  |  |  |  |  |  |  |  |  |  |  |  |  |  |  |  |  |  |
|  |  |  |  |  |  |  |  |  |  |  |  |  |  |  |  |  |  |  |  |  |  |  |  |  |  |  |  |  |  |  |  |  |  |  |  |  |  |  |  |  |  |  |  |  |
|  |  |  |  |  |  |  |  |  |  |  |  |  |  |  |  |  |  |  |  |  |  |  |  |  |  |  |  |  |  |  |  |  |  |  |  |  |  |  |  |  |  |  |  |  |
|  |  |  |  |  |  |  |  |  |  |  |  |  |  |  |  |  |  |  |  |  |  |  |  |  |  |  |  |  |  |  |  |  |  |  |  |  |  |  |  |  |  |  |  |  |
|  |  |  |  |  |  |  |  |  |  |  |  |  |  |  |  |  |  |  |  |  |  |  |  |  |  |  |  |  |  |  |  |  |  |  |  |  |  |  |  |  |  |  |  |  |
|  |  |  |  |  |  |  |  |  |  |  |  |  |  |  |  |  |  |  |  |  |  |  |  |  |  |  |  |  |  |  |  |  |  |  |  |  |  |  |  |  |  |  |  |  |
|  |  |  |  |  |  |  |  |  |  |  |  |  |  |  |  |  |  |  |  |  |  |  |  |  |  |  |  |  |  |  |  |  |  |  |  |  |  |  |  |  |  |  |  |  |
|  |  |  |  |  |  |  |  |  |  |  |  |  |  |  |  |  |  |  |  |  |  |  |  |  |  |  |  |  |  |  |  |  |  |  |  |  |  |  |  |  |  |  |  |  |
|  |  |  |  |  |  |  |  |  |  |  |  |  |  |  |  |  |  |  |  |  |  |  |  |  |  |  |  |  |  |  |  |  |  |  |  |  |  |  |  |  |  |  |  |  |
|  |  |  |  |  |  |  |  |  |  |  |  |  |  |  |  |  |  |  |  |  |  |  |  |  |  |  |  |  |  |  |  |  |  |  |  |  |  |  |  |  |  |  |  |  |

**Parameter: (Clinical Score = CS)**

Liveliness

- 0 Attentive, curious, gets up immediately
- 1 Somewhat tired, gets up hesitantly but independently
- 2 Listless, only gets up under force, immediately lies down again
- 3 Apathetic, cannot be convinced to get up despite stimulation

Posture

- 0 Relaxed posture, straight back
- 1 Stiff posture, arched back
- 2 Arched back, stiff movement over a longer period of time
- 3 Severely arched back or does not stand up

feeding behavior

- 0 Normal feeding behavior and weight gain, good musculature
- 1 Slightly reduced food intake, normal musculature
- 2 Anorexia, empty belly, weak musculature
- 3 Anorexia, sunken flanks, vertebrae and ribs visible, severe weight loss (over 15% of initial weight)

Respiration

- 0 Physiological, respiratory rate 10-15/min
- 1 Slightly increased respiratory rate, respiratory rate > 20/min
- 2 Moderately increased respiratory rate, respiratory rate > 20/min, clear respiratory movement
- 3 Dyspnea, greatly increased respiratory rate, respiratory rate > 30/min, mouth breathing

Movement

- 0 Coordinated movements
- 1 Unsteady movement, corrects crossed legs delayed
- 2 Significant ataxia/ weakness of hind legs, able to walk
- 3 Severe signs of paralysis, unable to walk

Lameness

- 0 Weight equally distributed on all four legs
- 1 Protection of one/several legs, shortened steps
- 2 Sparing of one/several legs, weight load on the leg is significantly shortened
- 3 Resting of one/several legs, can’t put weight on limbs

Skin

- 0 Evenly light pink, bristles not ruffled
- 1 Reddened skin areas
- 2 Blue-red discoloration, cold skin areas, individual punctiform hemorrhages
- 3 Black-blue discoloration, no sensitivity, extensive skin bleeding

Eyes/conjunctiva

- 0 Pale pink
- 1 Reddened, clear secretion
- 2 Very red, cloudy secretion
- 3 Very red, purulent secretion, vascular injection

Appetite

- 0 Greedy for offered food
- 1 Eats offered food hesitantly
- 2 Does not eat offered food, but sniffs at it
- 3 Eats nothing, no interest in food

Defecation

- 0 Formed feces, amount physiological
- 1 Mild diarrhea
- 2 Thin, mushy diarrhea
- 3 Watery to bloody diarrhea

Feed in the trough

- (not assessed for the individual animal)
- 0 Trough empty, cleanly eaten
- 1 Trough almost empty, little food left over
- 2 Feed only partially eaten, lots of feed left over
- 3 Trough still full, nothing eaten

The following criteria lead to closer monitoring (at least twice a day):

- Increase in rectal body temperature to over 40 °C
- Separation from the group
- Lack of interest in feeding/appetence
- Clinical score > 2 in one or more categories

If a cumulative CS > 5 is reached in an animal or a CS value of 3 is reached in a category, a veterinarian must be consulted immediately and treatment initiated if necessary.

Termination criteria:

Euthanasia under anesthesia is performed immediately if the total points of the CS reaches or exceeds 15 and treatment is not expected to be successful in the opinion of the attending veterinarian or curative treatment that has been initiated has not led to a significant reduction in clinical signs.

Animals with a low score but unexpectedly severe clinical signs that are not directly assessed with the clinical score are euthanized immediately (e.g. bone fractures, severe joint inflammation).

Definitive general termination criteria:

- Lack of food or fluid intake for more than 24 hours
- Decrease in body weight by > 20% compared to initial weight for more than 2 days
- Animal surface is cold; legs and abdominal skin bluish-dark as a sign of disturbed circulation in combination with shallow, strained breathing
- Severe mutilation (deep skin wounds, bitten toes, etc.)
- Severe central nervous deficits/stuck (e.g. symptoms of high-grade encephalitis such as spastic and central paralysis, clonic convulsions with uncoordinated forced movement and abnormal lying position
- Other signs suggesting an unacceptable condition

Specific termination criteria for hepatitis E in pigs

If signs of severe acute hepatitis/acute liver failure occur, a veterinarian is called in immediately to assess the clinical condition of the animal and the animal is monitored more closely (at least twice a day). If the symptoms are not reduced by 24 hours or if no significant reduction of signs is to be expected in the veterinarian's opinion, the animal has to be euthanized immediately. If more than two of the symptoms described below occur simultaneously, the animal must be removed from the experiment immediately and euthanized.

- Apathy (listlessness)
- Polydipsia (excessive binge drinking)
- Ascites (ascites)
- Icterus (jaundice)
- Vomitus (vomiting)
- Diarrhea
- Coagulation disorder
- Severe cramps
- Hepatoencephalic syndrome/central nervous deficits

## Supplemental Data 3

Code for statistical analysis in R (R Core Team [2023]. _R: A Language and Environment for Statistical Computing_. R Foundation for Statistical Computing, Vienna, Austria).

install.packages("tidyverse")

require(tidyverse)

#____________________________________________________

#Test for normal distribution:

my_data <- read.csv("K:/AG-INNT-Intern/Hrabal, Isabella/Vorträge_Berichte_Publikationen/Paper/5F6A1 Antikörperstudie/03 - results und Abbildungen/Statistik/V_nur_5F6A1.csv" ,sep=";")

shapiro.test(my_data$copies.µl.RNA.feces)

my_data$copies.µl.RNA.feces <- scale(my_data$copies.µl.RNA.feces)

qqnorm(my_data$copies.µl.RNA.feces)

qqline(my_data$copies.µl.RNA.feces)

hist(my_data$copies.µl.RNA.feces)

#--> nicht normal verteilt

#Logarithmieren und Test auf Normalverteilung:

my_data <- read.csv("K:/AG-INNT-Intern/Hrabal, Isabella/Vorträge_Berichte_Publikationen/Paper/5F6A1 Antikörperstudie/03 - results und Abbildungen/Statistik/V_nur_5F6A1.csv" ,sep=";")

my_data$copies.µl.RNA.feces <- log10(my_data$copies.µl.RNA.feces)

shapiro.test(my_data$copies.µl.RNA.feces)

my_data$copies.µl.RNA.feces <- scale(my_data$copies.µl.RNA.feces)

qqnorm(my_data$copies.µl.RNA.feces)

qqline(my_data$copies.µl.RNA.feces)

hist(my_data$copies.µl.RNA.feces)

#--> nicht normal verteilt

#Quadratwurzel und Test auf Normalverteilung:

my_data <- read.csv("K:/AG-INNT-Intern/Hrabal, Isabella/Vorträge_Berichte_Publikationen/Paper/5F6A1 Antikörperstudie/03 - results und Abbildungen/Statistik/V_nur_5F6A1.csv" ,sep=";")

my_data$copies.µl.RNA.feces <- sqrt(my_data$copies.µl.RNA.feces)

view(my_data)

shapiro.test(my_data$copies.µl.RNA.feces)

my_data$copies.µl.RNA.feces <- scale(my_data$copies.µl.RNA.feces)

qqnorm(my_data$copies.µl.RNA.feces)

qqline(my_data$copies.µl.RNA.feces)

hist(my_data$copies.µl.RNA.feces)

#--> nicht normal verteilt

#Kubikwurzel und Test auf Normalverteilung:

my_data <- read.csv("K:/AG-INNT-Intern/Hrabal, Isabella/Vorträge_Berichte_Publikationen/Paper/5F6A1 Antikörperstudie/03 - results und Abbildungen/Statistik/V_nur_5F6A1.csv" ,sep=";")

my_data$copies.µl.RNA.feces <- (my_data$copies.µl.RNA.feces)^(1/3)

view(my_data)

shapiro.test(my_data$copies.µl.RNA.feces)

my_data$copies.µl.RNA.feces <- scale(my_data$copies.µl.RNA.feces)

qqnorm(my_data$copies.µl.RNA.feces)

qqline(my_data$copies.µl.RNA.feces)

hist(my_data$copies.µl.RNA.feces)

#--> nicht normal verteilt

#______________________________________________________

#Normalverteilung nach logarithmieren nur Kontrollgruppe

#Test auf Normalverteilung:

my_data <- read.csv("K:/AG-INNT-Intern/Hrabal, Isabella/Vorträge_Berichte_Publikationen/Paper/5F6A1 Antikörperstudie/03 - results und Abbildungen/Statistik/V_nur_infektionskontrolle.csv" ,sep=";")

shapiro.test(my_data$copies.µl.RNA.feces)

my_data$copies.µl.RNA.feces <- scale(my_data$copies.µl.RNA.feces)

qqnorm(my_data$copies.µl.RNA.feces)

qqline(my_data$copies.µl.RNA.feces)

hist(my_data$copies.µl.RNA.feces)

#--> nicht normal verteilt

#Logarithmieren und Test auf Normalverteilung:

my_data <- read.csv("K:/AG-INNT-Intern/Hrabal, Isabella/Vorträge_Berichte_Publikationen/Paper/5F6A1 Antikörperstudie/03 - results und Abbildungen/Statistik/V_nur_infektionskontrolle.csv" ,sep=";")

my_data$copies.µl.RNA.feces <- log10(my_data$copies.µl.RNA.feces)

view(my_data)

shapiro.test(my_data$copies.µl.RNA.feces)

my_data$copies.µl.RNA.feces <- scale(my_data$copies.µl.RNA.feces)

qqnorm(my_data$copies.µl.RNA.feces)

qqline(my_data$copies.µl.RNA.feces)

hist(my_data$copies.µl.RNA.feces)

#--> nicht normal verteilt

#_____________________________________________________________________________

# Statistik T-Test Kot

my_data <- read.csv("K:/AG-INNT-Intern/Saskia Weber/13 - TV Schweine HEV/11 - Laboruntersuchungen Ergebnisse/04 - Teilversuch V - Antikörper/GesamtübersichtV.csv",sep=";")

my_data$copies.µl.RNA.feces <- replace(my_data$copies.µl.RNA.feces, my_data$copies.µl.RNA.feces <1, 0)

my_data$copies.µl.RNA.serum <- replace(my_data$copies.µl.RNA.serum, my_data$copies.µl.RNA.serum <1, 0)

my_data$brain <- replace(my_data$brain, my_data$brain <1, 0)

my_data$gallbladder <- replace(my_data$gallbladder, my_data$gallbladder <1, 0)

my_data$liver <- replace(my_data$liver, my_data$liver <1, 0)

my_data$spleen <- replace(my_data$spleen, my_data$spleen <1, 0)

my_data$kidney <- replace(my_data$kidney, my_data$kidney <1, 0)

my_data$Ln.Mes. <- replace(my_data$Ln.Mes., my_data$Ln.Mes. <1, 0)

my_data$bile <- replace(my_data$bile, my_data$bile <1, 0)

#statistik Kot gegen Infektionskontrolle

my_data %>%

filter(group %in% c("5F6A1 treatment", "infected control")) %>%

t.test(copies.µl.RNA.feces ~ group, data = .,

alternative = "two.sided")

#p-value = 5.769e-05

my_data %>%

filter(number %in% c("pig number 8", "pig number 16", "pig number 18","pig number 20","pig number 83", "pig number 92", "pig number 84", "pig number 88", "pig number 89", "pig number 90", "pig number 99", "pig number 12w")) %>%

filter(group %in% c("5F6A1 treatment", "infected control")) %>%

t.test(copies.µl.RNA.feces ~ group, data = .,

alternative = "less", #Alternative zur Nullhypotese heißt, ist treatmentkontrolle niedriger als infektionskontrolle

conf.level= 0.95)#statistik Kot gegen Infektionskontrolle

#p-value = 3.685e-05

# male vs female

my_data %>%

filter(number %in% c("pig number 8", "pig number 16")) %>%

filter(group %in% c("5F6A1 treatment", "infected control")) %>%

t.test(copies.µl.RNA.feces ~ number, data = .,

alternative = "less",

conf.level= 0.95)

#p-value = 0.6994

my_data %>%

filter(number %in% c("pig number 8", "pig number 16")) %>%

filter(group %in% c("5F6A1 treatment", "infected control")) %>%

t.test(copies.µl.RNA.feces ~ number, data = .,

alternative = "greater",

conf.level= 0.95)

#0-value = 0.3006

my_data %>%

filter(number %in% c("pig number 18", "pig number 16")) %>%

filter(group %in% c("5F6A1 treatment", "infected control")) %>%

t.test(copies.µl.RNA.feces ~ number, data = .,

alternative = "less",

conf.level= 0.95)

#p-value = 0.9432

my_data %>%

filter(number %in% c("pig number 18", "pig number 16")) %>%

filter(group %in% c("5F6A1 treatment", "infected control")) %>%

t.test(copies.µl.RNA.feces ~ number, data = .,

alternative = "greater",

conf.level= 0.95)

#p-value = 0.05679

my_data %>%

filter(number %in% c("pig number 20", "pig number 16")) %>%

filter(group %in% c("5F6A1 treatment", "infected control")) %>%

t.test(copies.µl.RNA.feces ~ number, data = .,

alternative = "less",

conf.level= 0.95)

#p-value = 0.8907

my_data %>%

filter(number %in% c("pig number 20", "pig number 16")) %>%

filter(group %in% c("5F6A1 treatment", "infected control")) %>%

t.test(copies.µl.RNA.feces ~ number, data = .,

alternative = "greater",

conf.level= 0.95)

#p-value = 0.1093

my_data %>%

filter(number %in% c("pig number 8", "pig number 18", "pig number 20", "pig number 16")) %>%

filter(group %in% c("5F6A1 treatment", "infected control")) %>%

filter(gender %in% c("f", "m")) %>%

t.test(copies.µl.RNA.feces ~ gender, data = .,

alternative = "less",

conf.level= 0.95)

#p-value = 0.1211

my_data %>%

filter(number %in% c("pig number 8", "pig number 18", "pig number 20", "pig number 16")) %>%

filter(group %in% c("5F6A1 treatment", "infected control")) %>%

filter(gender %in% c("f", "m")) %>%

t.test(copies.µl.RNA.feces ~ gender, data = .,

alternative = "greater",

conf.level= 0.95)

#p-value = 0.8789

my_data %>%

filter(number %in% c("pig number 8", "pig number 18", "pig number 20", "pig number 16")) %>%

filter(group %in% c("5F6A1 treatment", "infected control")) %>%

filter(gender %in% c("f", "m")) %>%

t.test(copies.µl.RNA.feces ~ gender, data = .,

alternative = "less",

conf.level= 0.95)

#p-value = 0.2422

my_data %>%

filter(number %in% c("pig number 8", "pig number 18", "pig number 20", "pig number 16")) %>%

filter(group %in% c("5F6A1 treatment", "infected control")) %>%

filter(gender %in% c("f", "m")) %>%

t.test(copies.µl.RNA.feces ~ gender, data = .,

alternative = "two.sided",

conf.level= 0.95)

#p-value = 0.2422

#__________________________________________________________________________

# Statistik Wilkoxon Test Kot

#Wilcoxon-Test/Mann-Whitney-U-Test

my_data <- read.csv("K:/AG-INNT-Intern/Hrabal, Isabella/Vorträge_Berichte_Publikationen/Paper/5F6A1 Antikörperstudie/03 - results und Abbildungen/Statistik/GesamtübersichtV_Wilcoxon_Organe.csv",sep=";") # sep = er nimmt ; als trennzeichen in der csv Dateo

my_data$copies.µl.RNA.feces <- replace(my_data$copies.µl.RNA.feces, my_data$copies.µl.RNA.feces <1, 0)

my_data$copies.µl.RNA.serum <- replace(my_data$copies.µl.RNA.serum, my_data$copies.µl.RNA.serum <1, 0)

my_data$brain <- replace(my_data$brain, my_data$brain <1, 0)

my_data$gallbladder <- replace(my_data$gallbladder, my_data$gallbladder <1, 0)

my_data$liver <- replace(my_data$liver, my_data$liver <1, 0)

my_data$spleen <- replace(my_data$spleen, my_data$spleen <1, 0)

my_data$kidney <- replace(my_data$kidney, my_data$kidney <1, 0)

my_data$Ln.Mes. <- replace(my_data$Ln.Mes., my_data$Ln.Mes. <1, 0)

my_data$bile <- replace(my_data$bile, my_data$bile <1, 0)

wilcox.test(my_data$copies.µl.RNA.feces ~ my_data$group.wilcox ,

exact = FALSE,

correct = FALSE,

conf.int = FALSE)

#W = 4485, p-value = 0.0006507

wilcox.test(my_data$copies.µl.RNA.feces ~ my_data$group.fm ,

exact = FALSE,

correct = FALSE,

conf.int = FALSE)

#W = 372.5, p-value = 0.3805

#################Statistik Serum###################

# Statistik T-Test Serum

my_data <- read.csv("K:/AG-INNT-Intern/Saskia Weber/13 - TV Schweine HEV/11 - Laboruntersuchungen Ergebnisse/04 - Teilversuch V - Antikörper/GesamtübersichtV.csv",sep=";")

#statistik Kot gegen Infektionskontrolle

my_data$copies.µl.RNA.feces <- replace(my_data$copies.µl.RNA.feces, my_data$copies.µl.RNA.feces <1, 0)

my_data$copies.µl.RNA.serum <- replace(my_data$copies.µl.RNA.serum, my_data$copies.µl.RNA.serum <1, 0)

my_data$brain <- replace(my_data$brain, my_data$brain <1, 0)

my_data$gallbladder <- replace(my_data$gallbladder, my_data$gallbladder <1, 0)

my_data$liver <- replace(my_data$liver, my_data$liver <1, 0)

my_data$spleen <- replace(my_data$spleen, my_data$spleen <1, 0)

my_data$kidney <- replace(my_data$kidney, my_data$kidney <1, 0)

my_data$Ln.Mes. <- replace(my_data$Ln.Mes., my_data$Ln.Mes. <1, 0)

my_data$bile <- replace(my_data$bile, my_data$bile <1, 0)

my_data %>%

filter(group %in% c("5F6A1 treatment", "infected control")) %>%

t.test(copies.µl.RNA.serum ~ group, data = .,

alternative = "two.sided")

#p-value = 0.0261

my_data %>%

filter(group %in% c("5F6A1 treatment", "infected control")) %>%

t.test(copies.µl.RNA.serum ~ group, data = .,

alternative = "less", #Alternative zur Nullhypotese heißt, ist treatmentkontrolle niedriger als infektionskontrolle

conf.level= 0.95)#statistik Kot gegen Infektionskontrolle

#p-value = 0.01305

#__________________________________________________________________________

# Statistik Wilkoxon Test Serum

#Wilcoxon-Test/Mann-Whitney-U-Test

my_data <- read.csv("K:/AG-INNT-Intern/Hrabal, Isabella/Vorträge_Berichte_Publikationen/Paper/5F6A1 Antikörperstudie/03 - results und Abbildungen/Statistik/GesamtübersichtV_Wilcoxon_Organe.csv",sep=";") # sep = er nimmt ; als trennzeichen in der csv Dateo

my_data$copies.µl.RNA.feces <- replace(my_data$copies.µl.RNA.feces, my_data$copies.µl.RNA.feces <1, 0)

my_data$copies.µl.RNA.serum <- replace(my_data$copies.µl.RNA.serum, my_data$copies.µl.RNA.serum <1, 0)

my_data$brain <- replace(my_data$brain, my_data$brain <1, 0)

my_data$gallbladder <- replace(my_data$gallbladder, my_data$gallbladder <1, 0)

my_data$liver <- replace(my_data$liver, my_data$liver <1, 0)

my_data$spleen <- replace(my_data$spleen, my_data$spleen <1, 0)

my_data$kidney <- replace(my_data$kidney, my_data$kidney <1, 0)

my_data$Ln.Mes. <- replace(my_data$Ln.Mes., my_data$Ln.Mes. <1, 0)

my_data$bile <- replace(my_data$bile, my_data$bile <1, 0)

wilcox.test(my_data$copies.µl.RNA.serum ~ my_data$group.wilcox ,

exact = FALSE,

correct = FALSE,

conf.int = FALSE)

#W = 1431, p-value = 0.00132

###############statistik liver ############

my_data <- read.csv("K:/AG-INNT-Intern/Saskia Weber/13 - TV Schweine HEV/11 - Laboruntersuchungen Ergebnisse/04 - Teilversuch V - Antikörper/GesamtübersichtV.csv",sep=";")

my_data$copies.µl.RNA.feces <- replace(my_data$copies.µl.RNA.feces, my_data$copies.µl.RNA.feces <1, 0)

my_data$copies.µl.RNA.serum <- replace(my_data$copies.µl.RNA.serum, my_data$copies.µl.RNA.serum <1, 0)

my_data$brain <- replace(my_data$brain, my_data$brain <1, 0)

my_data$gallbladder <- replace(my_data$gallbladder, my_data$gallbladder <1, 0)

my_data$liver <- replace(my_data$liver, my_data$liver <1, 0)

my_data$spleen <- replace(my_data$spleen, my_data$spleen <1, 0)

my_data$kidney <- replace(my_data$kidney, my_data$kidney <1, 0)

my_data$Ln.Mes. <- replace(my_data$Ln.Mes., my_data$Ln.Mes. <1, 0)

my_data$bile <- replace(my_data$bile, my_data$bile <1, 0)

my_data %>%

filter(group %in% c("5F6A1 treatment", "infected control")) %>%

t.test(liver ~ group, data = .,

alternative = "two.sided")

#p-value = 0.3846

my_data %>%

filter(group %in% c("5F6A1 treatment", "infected control")) %>%

t.test(liver ~ group, data = .,

alternative = "less")

# p-value = 0.1923

my_data <- read.csv("K:/AG-INNT-Intern/Hrabal, Isabella/Vorträge_Berichte_Publikationen/Paper/5F6A1 Antikörperstudie/03 - results und Abbildungen/Statistik/GesamtübersichtV_Wilcoxon_Organe.csv",sep=";") # sep = er nimmt ; als trennzeichen in der csv Dateo

my_data$copies.µl.RNA.feces <- replace(my_data$copies.µl.RNA.feces, my_data$copies.µl.RNA.feces <1, 0)

my_data$copies.µl.RNA.serum <- replace(my_data$copies.µl.RNA.serum, my_data$copies.µl.RNA.serum <1, 0)

my_data$brain <- replace(my_data$brain, my_data$brain <1, 0)

my_data$gallbladder <- replace(my_data$gallbladder, my_data$gallbladder <1, 0)

my_data$liver <- replace(my_data$liver, my_data$liver <1, 0)

my_data$spleen <- replace(my_data$spleen, my_data$spleen <1, 0)

my_data$kidney <- replace(my_data$kidney, my_data$kidney <1, 0)

my_data$Ln.Mes. <- replace(my_data$Ln.Mes., my_data$Ln.Mes. <1, 0)

my_data$bile <- replace(my_data$bile, my_data$bile <1, 0)

wilcox.test(my_data$liver ~ my_data$group.wilcox ,

exact = FALSE,

correct = FALSE,

conf.int = FALSE)

#W = 21, p-value = 0.06789

###############statistik gallbladder ############

my_data <- read.csv("K:/AG-INNT-Intern/Saskia Weber/13 - TV Schweine HEV/11 - Laboruntersuchungen Ergebnisse/04 - Teilversuch V - Antikörper/GesamtübersichtV.csv",sep=";")

my_data$copies.µl.RNA.feces <- replace(my_data$copies.µl.RNA.feces, my_data$copies.µl.RNA.feces <1, 0)

my_data$copies.µl.RNA.serum <- replace(my_data$copies.µl.RNA.serum, my_data$copies.µl.RNA.serum <1, 0)

my_data$brain <- replace(my_data$brain, my_data$brain <1, 0)

my_data$gallbladder <- replace(my_data$gallbladder, my_data$gallbladder <1, 0)

my_data$liver <- replace(my_data$liver, my_data$liver <1, 0)

my_data$spleen <- replace(my_data$spleen, my_data$spleen <1, 0)

my_data$kidney <- replace(my_data$kidney, my_data$kidney <1, 0)

my_data$Ln.Mes. <- replace(my_data$Ln.Mes., my_data$Ln.Mes. <1, 0)

my_data$bile <- replace(my_data$bile, my_data$bile <1, 0)

my_data %>%

filter(group %in% c("5F6A1 treatment", "infected control")) %>%

t.test(gallbladder ~ group, data = .,

alternative = "two.sided")

#p-value = 0.391

my_data %>%

filter(group %in% c("5F6A1 treatment", "infected control")) %>%

t.test(gallbladder ~ group, data = .,

alternative = "less")

#p-value = 0.1955

# Statistik Wilkoxon Test Kot

#Wilcoxon-Test/Mann-Whitney-U-Test

my_data <- read.csv("K:/AG-INNT-Intern/Hrabal, Isabella/Vorträge_Berichte_Publikationen/Paper/5F6A1 Antikörperstudie/03 - results und Abbildungen/Statistik/GesamtübersichtV_Wilcoxon_Organe.csv",sep=";") # sep = er nimmt ; als trennzeichen in der csv Dateo

my_data$copies.µl.RNA.feces <- replace(my_data$copies.µl.RNA.feces, my_data$copies.µl.RNA.feces <1, 0)

my_data$copies.µl.RNA.serum <- replace(my_data$copies.µl.RNA.serum, my_data$copies.µl.RNA.serum <1, 0)

my_data$brain <- replace(my_data$brain, my_data$brain <1, 0)

my_data$gallbladder <- replace(my_data$gallbladder, my_data$gallbladder <1, 0)

my_data$liver <- replace(my_data$liver, my_data$liver <1, 0)

my_data$spleen <- replace(my_data$spleen, my_data$spleen <1, 0)

my_data$kidney <- replace(my_data$kidney, my_data$kidney <1, 0)

my_data$Ln.Mes. <- replace(my_data$Ln.Mes., my_data$Ln.Mes. <1, 0)

my_data$bile <- replace(my_data$bile, my_data$bile <1, 0)

wilcox.test(my_data$gallbladder ~ my_data$group.wilcox ,

exact = FALSE,

correct = FALSE,

conf.int = FALSE)

#W = 15, p-value = 0.2207

###############statistik spleen ############

my_data <- read.csv("K:/AG-INNT-Intern/Saskia Weber/13 - TV Schweine HEV/11 - Laboruntersuchungen Ergebnisse/04 - Teilversuch V - Antikörper/GesamtübersichtV.csv",sep=";")

my_data$copies.µl.RNA.feces <- replace(my_data$copies.µl.RNA.feces, my_data$copies.µl.RNA.feces <1, 0)

my_data$copies.µl.RNA.serum <- replace(my_data$copies.µl.RNA.serum, my_data$copies.µl.RNA.serum <1, 0)

my_data$brain <- replace(my_data$brain, my_data$brain <1, 0)

my_data$gallbladder <- replace(my_data$gallbladder, my_data$gallbladder <1, 0)

my_data$liver <- replace(my_data$liver, my_data$liver <1, 0)

my_data$spleen <- replace(my_data$spleen, my_data$spleen <1, 0)

my_data$kidney <- replace(my_data$kidney, my_data$kidney <1, 0)

my_data$Ln.Mes. <- replace(my_data$Ln.Mes., my_data$Ln.Mes. <1, 0)

my_data$bile <- replace(my_data$bile, my_data$bile <1, 0)

my_data %>%

filter(group %in% c("5F6A1 treatment", "infected control")) %>%

t.test(spleen ~ group, data = .,

alternative = "two.sided")

#p-value = 0.2028

my_data %>%

filter(group %in% c("5F6A1 treatment", "infected control")) %>%

t.test(spleen ~ group, data = .,

alternative = "less")

#p-value = 0.1014

# Statistik Wilkoxon Test Kot

#Wilcoxon-Test/Mann-Whitney-U-Test

my_data <- read.csv("K:/AG-INNT-Intern/Hrabal, Isabella/Vorträge_Berichte_Publikationen/Paper/5F6A1 Antikörperstudie/03 - results und Abbildungen/Statistik/GesamtübersichtV_Wilcoxon_Organe.csv",sep=";") # sep = er nimmt ; als trennzeichen in der csv Dateo

my_data$copies.µl.RNA.feces <- replace(my_data$copies.µl.RNA.feces, my_data$copies.µl.RNA.feces <1, 0)

my_data$copies.µl.RNA.serum <- replace(my_data$copies.µl.RNA.serum, my_data$copies.µl.RNA.serum <1, 0)

my_data$brain <- replace(my_data$brain, my_data$brain <1, 0)

my_data$gallbladder <- replace(my_data$gallbladder, my_data$gallbladder <1, 0)

my_data$liver <- replace(my_data$liver, my_data$liver <1, 0)

my_data$spleen <- replace(my_data$spleen, my_data$spleen <1, 0)

my_data$kidney <- replace(my_data$kidney, my_data$kidney <1, 0)

my_data$Ln.Mes. <- replace(my_data$Ln.Mes., my_data$Ln.Mes. <1, 0)

my_data$bile <- replace(my_data$bile, my_data$bile <1, 0)

wilcox.test(my_data$spleen ~ my_data$group.wilcox ,

exact = FALSE,

correct = FALSE,

conf.int = FALSE)

#W = 18, p-value = 0.06789

###############statistik bile ############

my_data <- read.csv("K:/AG-INNT-Intern/Saskia Weber/13 - TV Schweine HEV/11 - Laboruntersuchungen Ergebnisse/04 - Teilversuch V - Antikörper/GesamtübersichtV.csv",sep=";")

my_data$copies.µl.RNA.feces <- replace(my_data$copies.µl.RNA.feces, my_data$copies.µl.RNA.feces <1, 0)

my_data$copies.µl.RNA.serum <- replace(my_data$copies.µl.RNA.serum, my_data$copies.µl.RNA.serum <1, 0)

my_data$brain <- replace(my_data$brain, my_data$brain <1, 0)

my_data$gallbladder <- replace(my_data$gallbladder, my_data$gallbladder <1, 0)

my_data$liver <- replace(my_data$liver, my_data$liver <1, 0)

my_data$spleen <- replace(my_data$spleen, my_data$spleen <1, 0)

my_data$kidney <- replace(my_data$kidney, my_data$kidney <1, 0)

my_data$Ln.Mes. <- replace(my_data$Ln.Mes., my_data$Ln.Mes. <1, 0)

my_data$bile <- replace(my_data$bile, my_data$bile <1, 0)

my_data %>%

filter(group %in% c("5F6A1 treatment", "infected control")) %>%

t.test(bile ~ group, data = .,

alternative = "two.sided")

#p-value = 0.3796

my_data %>%

filter(group %in% c("5F6A1 treatment", "infected control")) %>%

t.test(bile ~ group, data = .,

alternative = "less")

#p-value = 0.1898

# Statistik Wilkoxon Test Galle

#Wilcoxon-Test/Mann-Whitney-U-Test

my_data <- read.csv("K:/AG-INNT-Intern/Hrabal, Isabella/Vorträge_Berichte_Publikationen/Paper/5F6A1 Antikörperstudie/03 - results und Abbildungen/Statistik/GesamtübersichtV_Wilcoxon_Organe.csv",sep=";") # sep = er nimmt ; als trennzeichen in der csv Dateo

my_data$copies.µl.RNA.feces <- replace(my_data$copies.µl.RNA.feces, my_data$copies.µl.RNA.feces <1, 0)

my_data$copies.µl.RNA.serum <- replace(my_data$copies.µl.RNA.serum, my_data$copies.µl.RNA.serum <1, 0)

my_data$brain <- replace(my_data$brain, my_data$brain <1, 0)

my_data$gallbladder <- replace(my_data$gallbladder, my_data$gallbladder <1, 0)

my_data$liver <- replace(my_data$liver, my_data$liver <1, 0)

my_data$spleen <- replace(my_data$spleen, my_data$spleen <1, 0)

my_data$kidney <- replace(my_data$kidney, my_data$kidney <1, 0)

my_data$Ln.Mes. <- replace(my_data$Ln.Mes., my_data$Ln.Mes. <1, 0)

my_data$bile <- replace(my_data$bile, my_data$bile <1, 0)

wilcox.test(my_data$bile ~ my_data$group.wilcox ,

exact = FALSE,

correct = FALSE,

conf.int = FALSE)

#W = 22, p-value = 0.03248

References

1. Dähnert, L. *et al.* Immunisation of pigs with recombinant HEV vaccines does not protect from infection with HEV genotype 3. *One Health* **18,** 100674 (2024).

2. Mroz, C., Schmidt, K. M., Reiche, S., Groschup, M. H. & Eiden, M. Development of monoclonal antibodies to Rift Valley Fever Virus and their application in antigen detection and indirect immunofluorescence. *Journal of immunological methods* **460,** 36–44 (2018).
